# Analysis of circular RNA (circRNA) characteristics and identification of key circRNAs in the hypothalamus during sexual maturation in female goats

**DOI:** 10.5713/ab.25.0275

**Published:** 2025-06-24

**Authors:** Qing Li, Jianmin Wang, Yanyan Wang, Peipei He, Lu Zhang, Tianle Chao

**Affiliations:** 1 Shandong Provincial Key Laboratory of Animal Biotechnology and Disease Control and Prevention, College of Animal Science and Veterinary Medicine, Shandong Agricultural University, Tai’an, China; 2 Key Laboratory of Efficient Utilization of Non-grain Feed Resources (Co-construction by Ministry and Province), Ministry of Agriculture and Rural Affairs, Shandong Agricultural University, Tai’an, China

**Keywords:** Circular RNA, Competitive Endogenous RNA, Estrogen Signaling Pathway, Goat, Hypothalamus, Precocious Puberty

## Abstract

**Objective:**

Precocious puberty can shorten reproductive cycles, enhance reproductive capacity, and reduce feeding costs. Consequently, precocious livestock are widely utilized in cross-breeding. This study aims to elucidate the key molecular mechanisms by which circular RNA (circRNA) regulates the sexual maturation of sexually precocious goats.

**Methods:**

In this study, we analyzed the circRNA expression profiles of hypothalamic tissue from Jining grey goats at four distinct postnatal developmental stages (1 day, 2 months, 4 months, 6 months).

**Results:**

A total of 23,993 circRNAs were identified across these stages, predominantly derived from exonic regions, with 1,052 circRNAs exhibiting differential expression. Additionally, competitive endogenous RNA (ceRNA) analysis suggested that novel_circ_0002274/chi-miR-197-5p/estrogen receptor 1 (*ESR1*) and novel_circ_0002274/chi-miR-30c-3p/oxytocin/neurophysin I prepropeptide (*OXT*) may regulate sexual maturation in goats via the estrogen signaling pathway and the oxytocin signaling pathway.

**Conclusion:**

This study contributes to understanding the function of circRNAs in hypothalamic regulation of sexual maturation and provides valuable insights for breeding superior goat breeds.

## INTRODUCTION

Sexual maturation is a crucial phase for achieving reproductive success, influenced by various factors including genetics, nutrition, and neuroendocrine signaling within the hypothalamic-pituitary-gonadal (HPG) axis [1]. The process of postnatal sexual development relies on dynamic interactions among the hypothalamus, anterior pituitary gland, and gonads, which maintain normal reproductive function through feedback mechanisms [2]. The hypothalamus plays a pivotal role in this process. Sexual maturation primarily results from a significant increase in the secretory activity of hypothalamic gonadotropin-releasing hormone (GnRH) neurons, leading to enhanced synthesis and secretion of pituitary luteinizing hormone (LH) and follicle-stimulating hormone (FSH) [3]. The stimulation of these hormones promotes gonadal maturation, increases steroid hormone secretion, and facilitates gamete formation, all of which are essential for acquiring reproductive capacity [4]. In addition to endocrine changes, alterations in the neuronal network of the hypothalamus during sexual maturation also influence GnRH secretion, creating conditions conducive to sexual maturity [5]. These changes are characterized by a reduction in inhibitory inputs from Kisspeptin, neurokinin B, and dynorphin (KNDy) neurons in the hypothalamus, alongside increased excitatory signaling, both of which are critical factors in the initiation of puberty [6].

Goats are a significant source of meat, dairy products, and fiber. However, their relatively low reproductive capacity hinders the development of animal husbandry [7]. A comprehensive study on the physiological and molecular mechanisms that regulate sexual development in goats is essential for promoting genetic improvement and breeding practices. The Jining grey goat is a distinguished local breed in China, characterized by strong environmental adaptability, robust disease resistance, and tolerance to rough feeding [8]. The arrival of puberty in this breed occurs earlier than in other goat breeds, such as Boer goats (5–6 months) [9], Inner Mongolia Cashmere goats (5–6 months) [10], and Anhui White goats (4.5–5 months) [11]. Due to its precocious puberty, year-round estrus, and high reproductive rate, the Jining grey goat is considered an ideal model for studying the mechanisms regulating reproduction [10].

Circular RNA (CircRNA) is a unique RNA type characterized by covalently closed circular molecules that regulate transcription and translation of mRNA through interactions with RNA-binding proteins and microRNAs, thereby affecting gene expression [12]. In recent years, the study of circRNAs in the regulation of reproductive function has garnered considerable attention. Research on circRNAs in ovarian granulosa cells during human aging suggests that circRNA may be involved in the regulation of ovarian steroid production [13]. Furthermore, research has shown that circRNAs in goat ovarian tissue are essential for regulating the shift from the follicular phase to the luteal phase throughout the estrous cycle [14]. CircAkap17b acts as a molecular sponge for miR-7 in rat pituitary cells, influencing FSHβ expression and facilitating FSH secretion [15]. Studies of the hypothalamus in goats with low fertility indicate that circRNAs may modulate the fecundity of hypothalamic hormones by regulating their secretion [16].

Currently, most studies on circRNA in the hypothalamus focus on comparing high- and low-fertility livestock breeds. Regarding the role of circRNAs during the sexual maturation process in goats after birth, little research has been conducted. To address this, we have examined hypothalamic tissues collected from Jining grey goats at four postnatal stages (1 day, 2 months, 4 months, and 6 months) to analyze circRNA expression. This work provides a foundation for further studies on the role of circRNA on reproductive regulation.

## MATERIALS AND METHODS

### Animal and sample collection

This study selected 20 Jining grey goats from the Jining Grey Goat Breeding Farm in Jiaxiang County, Shandong Province, China (35.6°N, 116.3°E). During the non-breeding period (January 2022), all hypothalamic tissue samples were collected on the same day. The experimental goats were at four distinct phases: 1 day old (neonatal, D1, n = 5; body weight [BW]: 2.08±0.11 kg), 2 months old (prepubertal, M2, n = 5; BW: 4.42±0.24 kg), 4 months old (sexual maturity, M4, n = 5; BW: 7.62±0.50 kg), and 6 months old (breeding period, M6, n = 5; BW: 8.82±0.53 kg). All goats were raised under identical environmental conditions. The D1 and M2 goats were housed with their dams, while the M4 and M6 goats were separated from their does and placed in the same barn but in different pens. Consistent drinking water, lighting, temperature, and feeding conditions were maintained throughout. The selected goats were in good health, free of disease, and had freely access to food and water. The D1 and M2 groups did not exhibit estrus, while diestrus in the M4 and M6 groups was confirmed through external genital examination. The goats were slaughtered on the same day following euthanasia by electrocution. Hypothalamic samples were collected according to the method of Sesti and Britt [17], followed by immediate freezing in liquid nitrogen and storage at −80°C.

### Library construction and sequencing

RNA was isolated from hypothalamic tissue samples using TRIzol reagent (Invitrogen) according to the protocol provided by the manufacturer. To ensure the absence of genomic DNA, the extracted samples were treated with RNase-free DNase (Qiagen). The purity and integrity of the extracted RNA were measured using an Agilent 2100 Bioanalyzer, while the RNA concentration was determined with a NanoDrop ND-1000 spectrophotometer.

A circular RNA library was generated using a linear amplification-free approach. First, rRNA and linear RNA were depleted from the total RNA, and the remaining RNA was sheared into fragments of 250–300 base pair (bp). cDNA synthesis was conducted using the RNA fragments as templates, with random hexamers serving as primers during the process. The RNA strand was subsequently digested by RNase H. Second-strand cDNA was generated using DNA polymerase I and a dNTP mix. Following purification, the double-stranded cDNA was subjected to end repair, poly-A tailing, and adapter ligation. AMPure XP beads were utilized to isolate fragments within the 370–420 bp range. Following this step, the second-strand cDNA, which contained uracil, was treated with USER enzymes. The circular RNA library was then amplified via PCR. Sequencing of the prepared libraries was conducted on the Illumina NovaSeq 6000 platform, employing a paired-end read format of 150 base pairs.

### Identification of circular RNAs

High-quality reads were generated using fastp (v0.23.2), which filtered out adapter sequences, poly-N fragments, and low-quality segments. All downstream analyses utilized this high-quality dataset. The goat reference genome (GCF_ 001704415.2_ARS1.2) was indexed using HISAT2 (v2.0.5), with paired-end reads aligned to the reference genome.

Finally, circRNAs were detected and identified based on their structural features and splice site characteristics using find_circ and CIRI2 (v2.0.5). To quantify the expression levels of circRNAs accurately, the sequencing data were normalized using the transcripts per million (TPM) method. The TPM was calculated as: TPM = (read count×10^6) / total number of reads mapped to circRNAs.

### Differential expression analysis of circular RNA and functional enrichment analysis

Differentially expressed circRNAs (DECs) were determined with DESeq2 (v1.20.0) (p<0.05, |log2 fold change|>1). The expression profiles of DECs were analyzed using the MFUZZ package (v2.66.0). Enrichment analyses of GO and KEGG pathways were performed on the host genes of DECs exhibiting distinct expression patterns, utilizing the ClusterProfiler (v3.8.1) and KOBAS (v3.0.0). Terms and pathways with p<0.05 were considered significantly enriched.

### Competitive endogenous RNA network construction

The miRNA binding sites on DECs were identified using miRanda, while miRNA target genes were identified through a combination of miRanda (v3.3a) and RNAhybrid (v2.0). The circRNA-miRNA-mRNA network was constructed and visualized in Cytoscape (v3.4.0). Moreover, in conjunction with our previous findings [18], we subsequently assessed the correlation between key circRNAs within the competing endogenous RNA (ceRNA) network and serum hormone levels during the sexual development of goats.

### Verification of sequencing data

cDNA was synthesized with the PrimeScript RT Reagent Kit (Takara) per manufacturer’s protocol. Specific divergent primers were utilized to verify the circular characteristics of circRNA through a PCR-based approach. Utilizing the services of Sangon Biotech, Sanger sequencing analysis was conducted on the PCR-amplified products to detect back-splicing events. Subsequently, qRT-PCR reactions were performed on LightCycler 96 (Roche Diagnostics). A housekeeping gene, GAPDH, was employed for normalizing the data, with all qRT-PCR reactions conducted in three replicates. The 2^−ΔΔCt^ method was utilized for relative quantification of gene expression. Detailed information regarding the primer sequences employed in this investigation is provided in Supplement 1.

To validate the stability of circRNA, enzymatic digestion assays were performed using the RNase R Kit (Epicentre). Briefly, 5 μg of total RNA was treated with 20 U RNase R at 37°C for 35 minutes, followed by inactivation at 80°C for 10 minutes. This optimized protocol ensured the assessment of circRNA resistance to exonuclease digestion. Linear GAPDH served as an internal control, and its expression was quantitatively analyzed by qRT-PCR to evaluate circRNA stability. The data normalization process was conducted based on the expression levels of GAPDH mRNA.

## RESULTS

### Identification and analysis of circular RNAs in postnatal sexual maturation of hypothalamus in goats

To characterize the dynamic changes in the circRNA expression profile in the hypothalamic tissue of Jining grey goats, circRNA libraries were constructed across four developmental stages (n = 20). After quality control and filtering (Supplement 2), the average data volume for each sample was approximately 13.23 Gb, with Q20 exceeding 96.17% and Q30 exceeding 90.40%. Ultimately, we identified 23,993 circRNAs in the postnatal hypothalamic tissue of goats.

**Figure 1:**
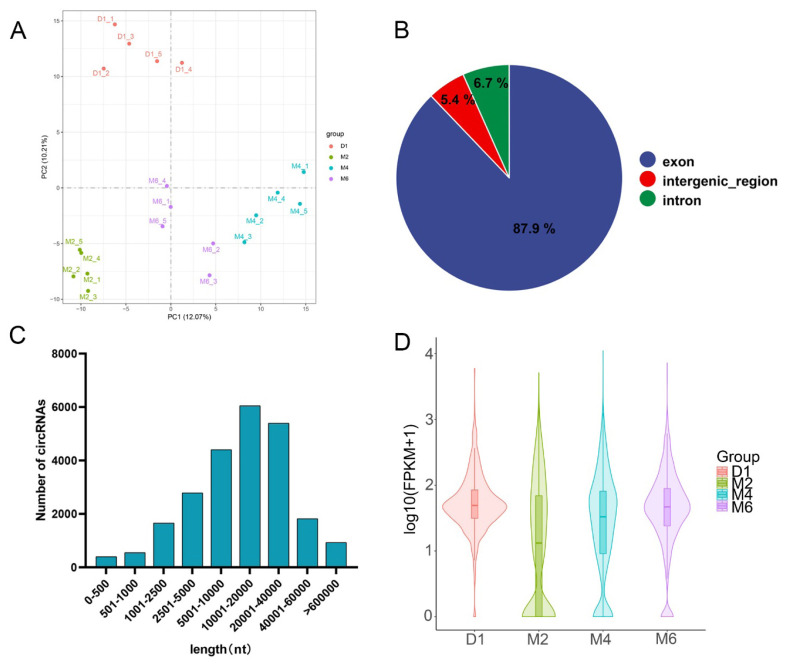
Identification and characterization of circular RNAs (circRNAs) in the hypothalamus of goats. (A) Principal component analysis (PCA) of circRNA expression in hypothalamic tissue. (B) Classification of circRNA types identified in the hypothalamus. (C) Length distribution of circRNAs detected in the hypothalamic tissue. (D) Box plots illustrating circRNA expression levels across different groups in the hypothalamus.

Principal component analysis (PCA) revealed a clear distinction in circRNA expression among different groups (Figure 1A). Feature analysis showed that the majority of circRNAs were derived from exons (87.8%), with the remainder originating from introns (6.7%) and intergenic regions (5.4%) (Figure 1B). The lengths of the identified circRNAs predominantly ranged from 5,000 bp to 40,000 bp (Figure 1C). Boxplots revealed significant differences in circRNA expression across groups (Figure 1D).

### Differential expression analysis of circular RNAs during hypothalamic sexual development

**Figure 2:**
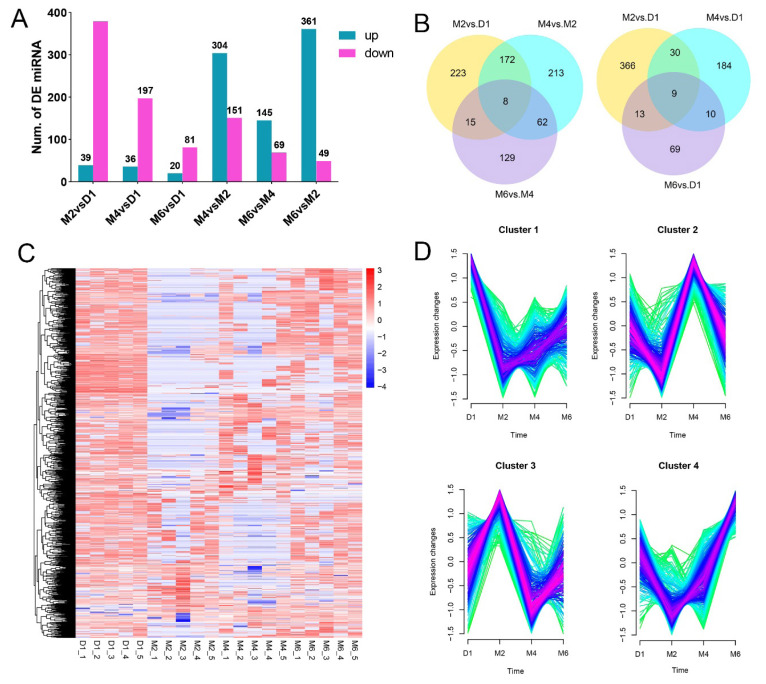
Differential expression analysis of circular RNAs (circRNAs) in the hypothalamus. (A) Bar chart depicting the distribution of differentially expressed circRNAs (DECs) in the hypothalamic tissue. (B) Heatmap of clustering analysis for DECs in the hypothalamus. (C) Venn diagram illustrating the common and unique DECs across different comparison groups in the hypothalamus. (D) Analysis of expression patterns for DECs in the hypothalamic tissue.

To compare circRNA expression patterns at different developmental stages in goats, differential expression analysis was performed using DESeq2, identifying a total of 1,052 DECs through pairwise comparisons (Figure 2A, Supplement 3). Specifically, 418 DECs (39 upregulated and 379 downregulated) were identified in M2 vs. D1; 233 DECs (36 upregulated and 197 downregulated) in M4 vs. D1; 101 DECs (20 upregulated and 81 downregulated) in M6 vs. D1; 455 DECs (304 upregulated and 151 downregulated) in M4 vs. M2; 214 DECs (145 upregulated and 69 downregulated) in M6 vs. M4; and 410 DECs (361 upregulated and 49 downregulated) in M6 vs. M2.

We further constructed Venn diagrams to illustrate the DECs identified in the different comparison groups. Compared to the D1 group, eight common DECs were identified in the M2, M4, and M6 groups (Figure 2B, Supplement 4A), specifically: novel_circ_0038172, 0039204, 0040081, 0033030, 0039865, 0008507, 0005958, and 0037875. Additionally, eight common DECs were identified in the comparisons of M2 vs. D1, M4 vs. M2, and M6 vs. M4 (Figure 2B, Supplement 4B), which included: novel_circ_0040957, 0021810, 0023262, 0015100, 0017010, 0016246, 0036328, and 0024220.

Hierarchical clustering analysis of circRNAs indicated that these molecules may participate in the post-birth sexual development of goats through specific expression patterns (Figure 2C). Further analysis revealed that the DECs identified in the hypothalamic tissue across the four developmental stages exhibited four distinct expression patterns (Figure 2D, Supplement 5). DECs in Cluster 1 displayed a significant decreasing trend from D1 to M2, followed by a gradual increase. DECs in Cluster 2 reached their highest expression levels during M4. DECs in Cluster 3 exhibited an initial increase followed by a decrease from D1 to M4, with a slight rebound by M6. DECs in Cluster 4 showed the lowest expression levels during M2, followed by an upward trend, peaking at M6.

### Functional enrichment analysis with differential expression of circular RNAs during hypothalamic sexual development

A total of 1,052 DECs were identified, originated from 799 unique genes, demonstrating that individual genes have the capacity to generate multiple circRNAs. Functional enrichment analysis was performed through GO and KEGG enrichment analyses targeting the host genes of DECs exhibiting distinct expression patterns (Supplements 6, 7).

**Figure 3:**
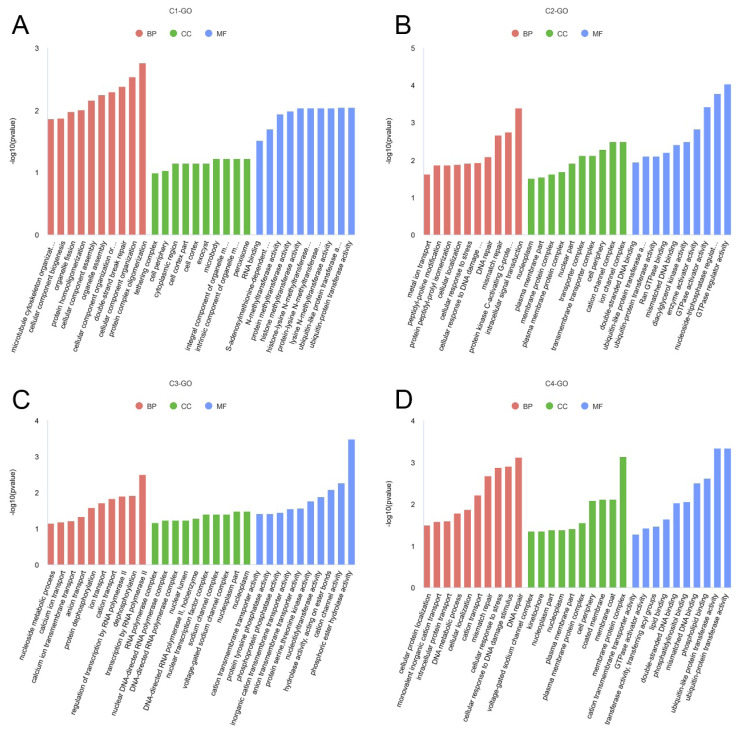
GO enrichment analysis of host genes for circular RNAs (circRNAs) with different expression patterns in the hypothalamus of goats. (A–D) GO enrichment analysis of host genes from circRNAs in Clusters 1 to 4. BP, biological process; CC, cellular component; MF, molecular function.

**Figure 4:**
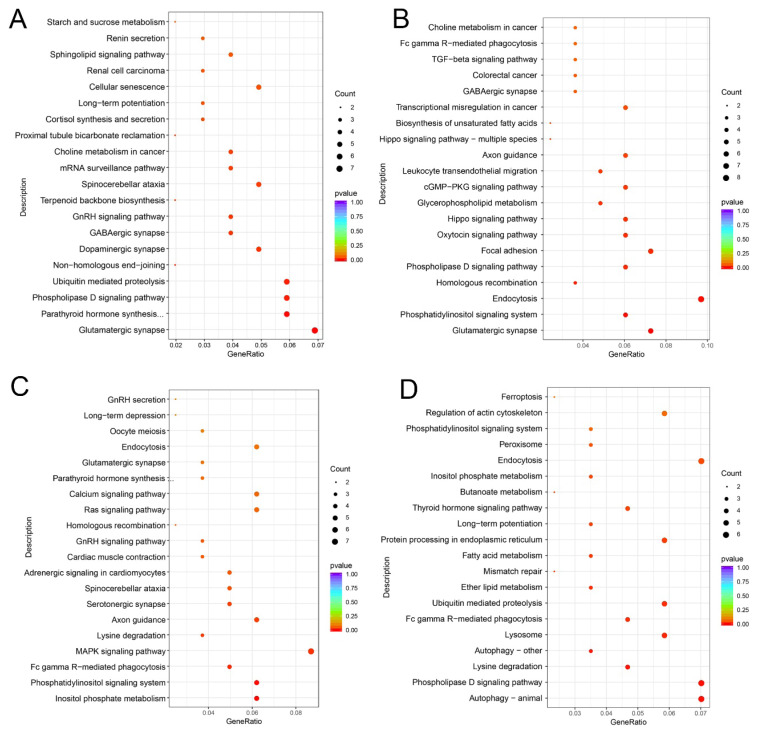
KEGG enrichment analysis of host genes for circular RNAs (circRNAs) with different expression patterns in the hypothalamus of goats. (A–D) KEGG enrichment analysis of host genes from circRNAs in Clusters 1 to 4.

For Cluster 1 DECs, the significantly enriched GO terms included protein complex oligomerization, and small GTPase mediated signal transduction (p<0.05) (Figure 3A). KEGG analysis revealed significant enrichment in pathways such as glutamatergic synapse, Parathyroid hormone synthesis, secretion and action, Phospholipase D signaling pathway, Dopaminergic synapse, GABAergic synapse, and GnRH signaling pathway (p<0.05) (Figure 4A). For Cluster 2 DECs, the significantly enriched GO terms were protein kinase C-activating G-protein-coupled receptor signaling pathway, and calcium channel activity (p<0.05) (Figure 3B). KEGG analysis indicated significant enrichment in pathways including Glutamatergic synapse, Phosphatidylinositol signaling system, and GABAergic synapse (p<0.05) (Figure 4B). For Cluster 3 DECs, the significantly enriched GO terms included transcription by RNA polymerase II and dephosphorylation (p< 0.05) (Figure 3C). KEGG analysis showed significant enrichment in pathways such as Inositol phosphate metabolism, MAPK signaling pathway, and GnRH signaling pathway (p< 0.05) (Figure 4C). For Cluster 4 DECs, the significantly enriched GO terms were ubiquitin-protein transferase activity, ubiquitin-like protein transferase activity, and phospholipid binding (p<0.05) (Figure 3D). KEGG analysis indicated significant enrichment in the Fatty acid metabolism, Thyroid hormone signaling pathway, and Inositol phosphate metabolism (p<0.05) (Figure 4D).

### Construction of competitive endogenous RNA network

**Figure 5:**
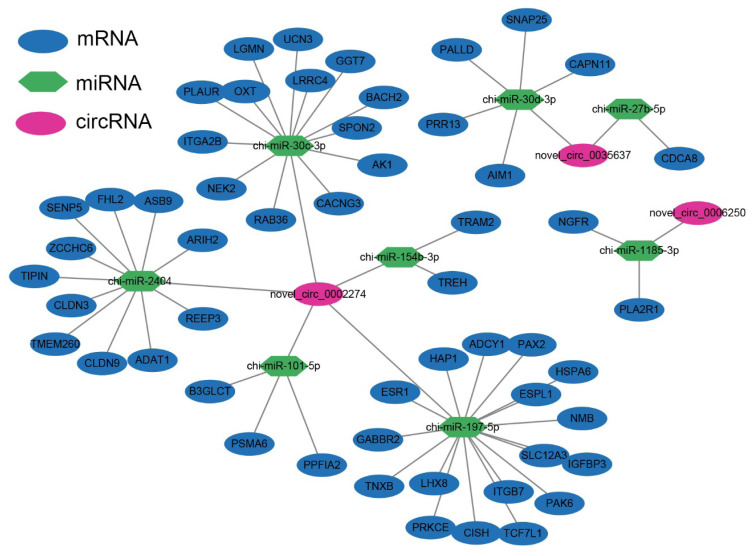
Construction of the circular RNA (circRNA)-related competitive endogenous RNA (ceRNA) regulatory network. Blue nodes represent mRNAs, pink nodes represent miRNAs, and green nodes represent circRNAs.

**Figure 6:**
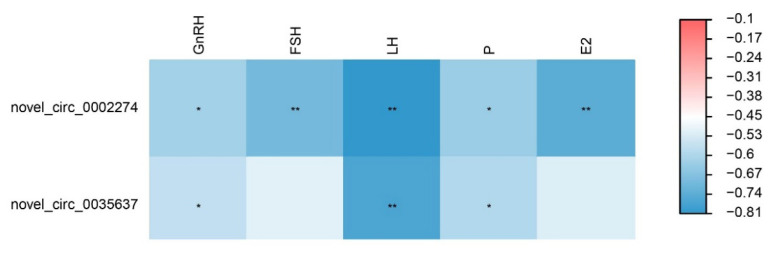
Correlation analysis between circRNAs and serum hormone levels in goats. Red indicates a positive correlation, while blue signifies a negative correlation. * p<0.05; ** p<0.01. GnRH, gonadotropin-releasing hormone; FSH, follicle-stimulating hormone; LH, luteinizing hormone; P, progesterone; E2, estradiol; circRNA, circular RNA.

To investigate the interactions between circRNAs, mRNAs, and miRNAs during postnatal sexual development in goats, we established a ceRNA regulatory network (Figure 5). Based on the ceRNA hypothesis, we identified a total of 85 ceRNA interactions, which comprised 15 DE miRNAs, 5 DECs, and 75 mRNAs. Notably, novel_circ_0002274 exhibited the most regulatory relationships, targeting chi-miR-30c-3p, chi-miR-154b-3p, chi-miR-101-5p, chi-miR-2404, and chi-miR-197-5p. Among these, chi-miR-197-5p had the highest number of targeted mRNAs (17). We further conducted KEGG analysis on the mRNAs within the ceRNA network. The results indicated significant enrichment in pathways associated with reproduction and signal transduction, including the Estrogen signaling pathway, Oxytocin signaling pathway, GABAergic synapse, PI3K-Akt signaling pathway, and Neuroactive ligand-receptor interaction (Supplements 8, 9). Additionally, we calculated the correlation between key circRNAs within the ceRNA network and serum hormone levels during the sexual maturation process in goats (Figure 6). Notably, both novel_circ_0002274 and novel_circ_0035637 exhibited significant negative correlations with GnRH, LH, and E2 (p<0.05). Furthermore, novel_circ_0035637 displayed a significant negative correlation with FSH (p<0.05). The results of the correlation analysis indicate that these circRNAs may regulate in hormone secretion during the sexual development of goats through the ceRNA network.

### Experimental verification of circular RNA

**Figure 7:**
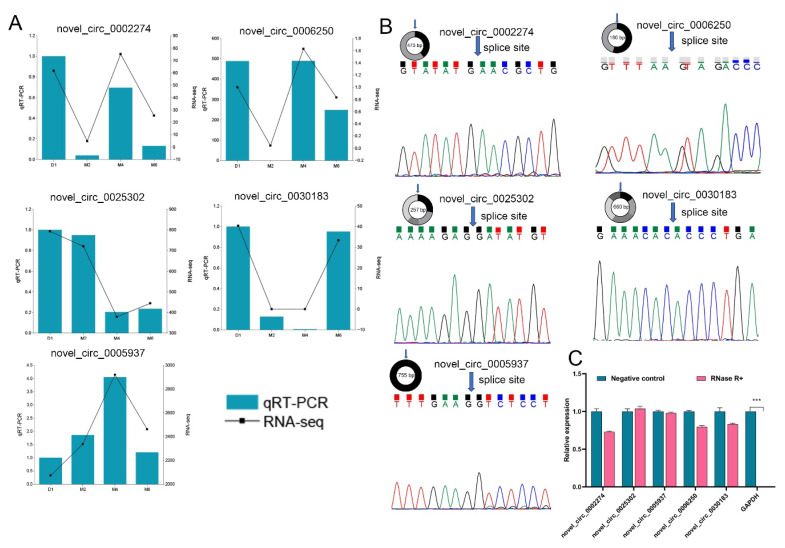
Validation of circular RNAs (circRNAs). (A) qRT-PCR validation of differentially expressed circRNAs at various developmental stages. (B) Sanger sequencing confirmed the back-splicing junction of circRNAs. (C) qRT-PCR validation of RNase R digestion assays, with linear GAPDH mRNA used as an internal control. All qRT-PCR experiments were performed in triplicate (n = 3). qRT-PCR, quantitative reverse transcription polymerase chain reaction. *** p<0.001.

To verify the reliability of RNA-Seq data, we selected five DECs for qRT-PCR validation. The results indicated that the expression profiles of these five DECs were consistent with the trends observed in the RNA-Seq data (Figure 7A).

To further validate the circular structure of the circRNAs, we designed divergent primers that span the splice sites and performed PCR amplification, followed by Sanger sequencing. The sequencing results demonstrated (Figure 7B, Supplement 10) that the circRNAs indeed had back-splice junctions, confirming their circular structure. Additionally, the results from RNase R digestion assays showed (Figure 7C) that these circRNAs exhibited resistance to RNase R. These findings collectively indicate that the sequencing results of the circRNAs are reliable.

## DISCUSSION

Precocious puberty shortens the reproductive cycle and enhances reproductive capacity [19]. The hypothalamus serves as a key player in sexual development and reproductive processes by processing both intrinsic and extrinsic signals to modulate GnRH secretion, thereby governing gonadal function [20,21]. However, research on the molecular mechanisms by which circRNA regulates sexual maturation in the hypothalamic tissue of precocious goats remains limited.

This study identified 23,993 circRNAs across four developmental stages (D1, M2, M4, M6) in Jining grey goats. This number is lower than those reported in other goats hypothalamic circRNA studies [22,23], suggesting that circRNAs show both tissue specificity and temporal specificity. Among these, 1,052 DECs were detected. CircRNAs are generated through the back-splicing of linear RNAs, and their functions are closely related to their host genes [24]. Key DECs and their host genes were identified, with novel_circ_0021810 showing differential expression in the comparisons of M2 vs. D1, M4 vs. M2, and M6 vs. M4. Its host gene Homer scaffold protein 1 (*HOMER1*), a postsynaptic scaffolding protein predominantly expressed in the nervous system, mediates synaptic plasticity and intracellular signaling [25,26]. *HOMER1* also modulates glutamatergic receptor signaling in the hypothalamic suprachiasmatic nucleus (SCN) to regulate circadian rhythms [27,28]. Similarly, novel_circ_0021810 exhibited differential expression across M2 vs. D1, M4 vs. D1, and M6 vs. D1 comparisons. BCAS3, the host gene of novel_circ_0033030, has been identified as a candidate gene associated with goat reproduction [29].

To investigate the potential roles of circRNAs in sexual maturity, functional analysis was conducted on hosting genes of DECs. This analysis revealed significant enrichment in pathways including GABAergic neurotransmission, GnRH signaling, phosphatidylinositol signaling pathways, and dopaminergic neurotransmission. Notably, the phospholipase D signaling pathway was significantly enriched in clusters 1, 2, and 4. Similarly, glutamatergic and GABAergic synapses pathway were significantly enriched in clusters 1 and 2. Phospholipase D signaling critically regulates hypothalamic GnRH secretion. As a shared downstream effector of phospholipase C (PLC) and voltage-gated pathways in GnRH neurons [30], Phospholipase D mobilizes intracellular Ca^2+^ and activates PKC, triggering MAPK/ERK cascades that modulate pituitary gonadotropin release [31]. Phospholipase D1 (PLD1) additionally mediates vesicle trafficking, secretion, and receptor signaling [32], while contributing to metabolic homeostasis [33]. We identified novel_circ_0002691, novel_circ_0002655, and novel_circ_0002694 as *PLD1* derived circRNAs. Significantly, novel_circ_0002655 and novel_circ_0002694 showed elevated expression during M4/M6 developmental stages, implicating their role in *PLD1* dependent hypothalamic GnRH regulation. Glutamatergic and GABAergic inputs critically regulate GnRH secretion and sexual maturation [34,35]. In sheep, estrogen fluctuations during the follicular phase drive glutamate-dependent synaptic remodeling in KNDy neurons [36]. This plasticity enhances GnRH neuron sensitivity to glutamate while remodeling KNDy neuronal density, promoting pulsatile GnRH/LH secretion and preovulatory LH surge generation [37]. We identified glutamate receptor 5 (*GRM5*) as the host gene of novel_circ_0043162. *GRM5* mediates glutamatergic neurotransmission and contributes to glucose homeostasis via hypothalamic PACAP neuron modulation [38,39]. The scaffolding protein Homer1 functionally enhances *GRM5* activity [40]. Our findings suggest novel_circ_ 0043162 and novel_circ_0021810 may coordinately modulate hypothalamic neuronal signaling during caprine sexual maturation by targeting host genes *GRM5* and Homer1, respectively. GABAergic neurotransmission regulates the onset of puberty in female goats [41]. As a key component of GABAergic synapses pathway, adenylate cyclase 5 (*ADCY5*) modulates neurotrophic signaling in GnRH neurons of mice [42]. The biosynthesis of GABA in the brain depends on glutamate, produced by glutaminase (GLS) through glutamine conversion [43]. Changes in GLS mRNA levels in the hypothalamus can regulate GABAergic neurotransmission, influencing GnRH secretion [44]. In this study, we identified protein phosphatase 3 catalytic subunit α (*PPP3CA*) as the host gene of novel_circ_0012695. *PPP3CA* critically regulates in glutamatergic synapses and is a key regulator of puberty in cattle [45,46]. These findings suggest that novel_circ_0001964 and novel_circ_0012695, via their host genes *ADCY5* and *PPP3CA*, respectively, may coordinately regulate GnRH neuronal activity in the hypothalamus by modulating the interplay between GABAergic and glutamatergic systems, affecting the progression of puberty.

CircRNAs function as miRNA sponges to regulate mRNA expression through a ceRNA mechanism, thereby participating in reproductive regulation [47]. The mRNAs in the ceRNA network constructed in this study were significantly enriched in the estrogen signaling and oxytocin signaling pathways. Estrogen signaling pathway is known to play a crucial role in regulating growth and pubertal development in the hypothalamus [48]. Estrogen receptor α (*ESR1*), expressed in the hypothalamus, serves as a key receptor for estrogen in negative feedback on the gonadal axis, thereby regulating GnRH secretion [49]. Mice lacking Erα in hypothalamic kisspeptin neurons display precocious puberty without reaching sexual maturity or displaying normal ovulation cycles [50]. The oxytocin signaling pathway plays a significant role in reproductive regulation and sexual maturation in mammals [51,52]. Oxytocin/neurophysin I (*Oxt*) is widely expressed in hypothalamic tissues [53]. Oxytocin is a neuropeptide synthesized in the hypothalamus, stored in the posterior pituitary, and released into the bloodstream; it acts as a neurotransmitter in various processes, including sexual development and maternal behavior [54]. Studies indicate that oxytocin can promote the maturation of female mice by facilitating GnRH release [55]. *CACNG3*, as a voltage-gated calcium channel (VGCC) γ subunit, may regulate GnRH secretion by influencing calcium ion influx in neurons [22]. These findings suggest that *CACNG3* and *OXT* genes play significant roles in hormone secretion regulation during goat sexual maturation by modulating hypothalamic neurons. Novel_circ_0002274 targets chi-miR-30c-3p, co-regulating the expression of *OXT* and *CACNG3*, and thus plays a crucial role in goat sexual maturation. In the identified ceRNA network, novel_circ_0002274 was predicted to act as a sponge for chi-miR-197-5p, which targets ESR1; additionally, novel_circ_0002274 was predicted to act as a sponge for chi-miR-30c-3p, which targets to *CACNG3* and *OXT*. In this study, the expression levels of novel_circ_0002274 showed a significant negative correlation with the serum concentrations of GnRH, FSH, LH, and E2 throughout the postnatal sexual maturation process in goats postnatally. In summary, novel_circ_0002274 may play an essential role in regulating sexual maturation in the hypothalamus via the estrogen and oxytocin signaling pathways.

This study has certain limitations. While key circRNAs and ceRNA regulatory networks were identified through bioinformatics analyses, the specific molecular mechanisms by which these circRNAs regulate goat sexual development require experimental validation. Additionally, this research focused on precocious goat breeds, and further validation is needed to determine its applicability to other goat breeds.

This study has certain limitations. Although we have conducted preliminary validation of the structures of some circRNAs, the functional roles of the key circRNAs and their ceRNA networks in regulating the sexual maturation process of goats, identified primarily through bioinformatics analyses such as KEGG pathway enrichment, remain to be substantiated by further molecular biological experiments. Additionally, this research focused on precocious goat breeds, and further validation is needed to determine its applicability to other goat breeds.

## CONCLUSION

This study characterizes the dynamic expression profile of circRNAs in the hypothalamic tissue of Jining grey goats during sexual maturation. The novel_circ_0002655, novel_circ_ 0043162, novel_circ_0001964, and novel_circ_0012695, along with their respective host genes—*PLD1*, *GRM5*, *ADCY5*, and *PPP3CA*—may regulate hypothalamic neuronal signal transduction and GnRH secretion by participating in crucial pathways such as phospholipase D signaling, glutamatergic synaptic transmission, and GABAergic synapse modulation. Furthermore, the novel_circ_0002274/chi-miR-197-5p/*ESR1* and novel_circ_0002274/chi-miR-30c-3p/*OXT* axes may constitute pivotal ceRNA networks, regulating goat sexual maturation and its feedback mechanisms on the HPG axis. These results provide new insights into the circRNA expression profiles in the goat hypothalamus and lay the foundation for exploring hypothalamic regulatory mechanisms influences sexual development in goats.

## SUPPLEMENTARY MATERIAL

**Supplement 1.** Primers for circRNA validation and qPCR.

**Supplement 2.** Summary of reads mapped to the goat genome.

**Supplement 3.** Differentially expressed circRNA was identified during sexual development in goats.

**Supplement 4.** Key circRNA clustering heatmap.

**Supplement 5.** Different expression patterns of circRNA were identified by mfuzz.

**Supplement 6.** GO analysis of CircRNA-derived genes with different expression patterns.

**Supplement 7.** KEGG analysis of CircrNA-derived genes with different expression patterns.

**Supplement 8.** KEGG enrichment analysis of mRNAs in the ceRNA network.

**Supplement 9.** KEGG analysis of mrna in ceRNA network.

**Supplement 10.** circRNA junction sequence information.

## References

[1] Plant TM (2015). 60 Years of neuroendocrinology: the hypothalamo-pituitary–gonadal axis. J Endocrinol.

[2] Avendaño MS, Vazquez MJ, Tena-Sempere M (2017). Disentangling puberty: novel neuroendocrine pathways and mechanisms for the control of mammalian puberty. Hum Reprod Update.

[3] Herbison A (2016). Control of puberty onset and fertility by gonadotropin-releasing hormone neurons. Nat Rev Endocrinol.

[4] Lomniczi A, Ojeda SR, Bourguignon JP, Parent AS (2015). The emerging role of epigenetics in the regulation of female puberty. Puberty from bench to clinic: lessons for clinical management of pubertal disorders.

[5] Livadas S, Chrousos GP (2016). Control of the onset of puberty. Curr Opin Pediatr.

[6] Prague JK, Dhillo WS (2015). Potential clinical use of kisspeptin. Neuroendocrinology.

[7] Gawat M, Boland M, Singh J, Kaur L (2023). Goat meat: production and quality attributes. Foods.

[8] Liu G, Zhao Q, Lu J (2019). Insights into the genetic diversity of indigenous goats and their conservation priorities. Asian-Australas J Anim Sci.

[9] Greyling JPC (2000). Reproduction traits in the Boer goat doe. Small Rumin Res.

[10] Cao GL, Chu MX, Fang L, Di R, Feng T, Li N (2010). Analysis on DNA sequence of KiSS-1 gene and its association with litter size in goats. Mol Biol Rep.

[11] Ye J, Yao Z, Si W (2018). Identification and characterization of microRNAs in the pituitary of pubescent goats. Reprod Biol Endocrinol.

[12] Chen LL (2016). The biogenesis and emerging roles of circular RNAs. Nat Rev Mol Cell Biol.

[13] Cheng J, Huang J, Yuan S (2017). Circular RNA expression profiling of human granulosa cells during maternal aging reveals novel transcripts associated with assisted reproductive technology outcomes. PLOS ONE.

[14] Liu Y, Zhou Z, He X (2022). Differentially expressed circular RNA profile signatures identified in prolificacy trait of Yunshang black goat ovary at estrus cycle. Front Physiol.

[15] Wang CJ, Gao F, Huang YJ (2020). circAkap17b acts as a miR-7 family molecular sponge to regulate FSH secretion in rat pituitary cells. J Mol Endocrinol.

[16] Mao S, Wu C, Feng G (2024). Selection and regulatory network analysis of differential CircRNAs in the hypothalamus of goats with high and low reproductive capacity. Int J Mol Sci.

[17] Sesti LAC, Britt JH (1993). Relationship of secretion of GnRH in vitro to changes in pituitary concentrations of LH and FSH and serum concentrations of LH during lactation in sows. J Reprod Fertil.

[18] Li Q, Chao T, Wang Y (2024). Comparative metabolomics reveals serum metabolites changes in goats during different developmental stages. Sci Rep.

[19] Cortés ME, Carrera B, Rioseco H, Pablo del Río J, Vigil P (2015). The role of kisspeptin in the onset of puberty and in the ovulatory mechanism: a mini-review. J Pediatr Adolesc Gynecol.

[20] Yang F, Zhao S, Wang P, Xiang W (2023). Hypothalamic neuroendocrine integration of reproduction and metabolism in mammals. J Endocrinol.

[21] Naulé L, Maione L, Kaiser UB (2021). Puberty, a sensitive window of hypothalamic development and plasticity. Endocrinology.

[22] Zhang XB, Spergel DJ (2012). Kisspeptin inhibits high-voltage activated Ca2+ channels in GnRH neurons via multiple Ca2+ influx and release pathways. Neuroendocrinology.

[23] Zheng Q, Bao C, Guo W (2016). Circular RNA profiling reveals an abundant circHIPK3 that regulates cell growth by sponging multiple miRNAs. Nat Commun.

[24] Hanan M, Soreq H, Kadener S (2017). CircRNAs in the brain. RNA Biol.

[25] Shiraishi-Yamaguchi Y, Furuichi T (2007). The Homer family proteins. Genome Biol.

[26] Luo P, Li X, Fei Z, Poon W (2012). Scaffold protein Homer 1: implications for neurological diseases. Neurochem Int.

[27] Nielsen HS, Georg B, Hannibal J, Fahrenkrug J (2002). Homer-1 mRNA in the rat suprachiasmatic nucleus is regulated differentially by the retinohypothalamic tract transmitters pituitary adenylate cyclase activating polypeptide and glutamate at time points where light phase-shifts the endogenous rhythm. Mol Brain Res.

[28] Wang Q, Chikina MD, Pincas H, Sealfon SC (2014). Homer1 alternative splicing is regulated by gonadotropin-releasing hormone and modulates gonadotropin gene expression. Mol Cell Biol.

[29] Liu Z, Tan X, Wang J (2022). Whole genome sequencing of Luxi black head sheep for screening selection signatures associated with important traits. Anim Biosci.

[30] Zheng L, Krsmanovic LZ, Vergara LA, Catt KJ, Stojilkovic SS (1997). Dependence of intracellular signaling and neurosecretion on phospholipase D activation in immortalized gonadotropin-releasing hormone neurons. Proc Natl Acad Sci USA.

[31] Naor Z, Harris D, Shacham S (1998). Mechanism of GnRH receptor signaling: combinatorial cross-talk of Ca2+ and protein kinase C. Front Neuroendocrinol.

[32] Riebeling C, Morris AJ, Shields D (2009). Phospholipase D in the Golgi apparatus. Biochim Biophys Acta Mol Cell Biol Lipids.

[33] Trujillo Viera J, El-Merahbi R, Nieswandt B, Stegner D, Sumara G (2016). Phospholipases D1 and D2 suppress appetite and protect against overweight. PLOS ONE.

[34] Herbison AE, Moenter SM (2011). Depolarising and hyperpolarising actions of GABAA receptor activation on gonadotrophin-releasing hormone neurones: towards an emerging consensus. J Neuroendocrinol.

[35] Jackson GL, Kuehl D (2002). Gamma-aminobutyric acid (GABA) regulation of GnRH secretion in sheep. Reprod Suppl.

[36] Porter DT, Goodman RL, Hileman SM, Lehman MN (2021). Evidence that synaptic plasticity of glutamatergic inputs onto KNDy neurones during the ovine follicular phase is dependent on increasing levels of oestradiol. J Neuroendocrinol.

[37] Goodman RL, Moore AM, Onslow K (2023). Lesions of KNDy and Kiss1R neurons in the arcuate nucleus produce different effects on LH pulse patterns in female sheep. Endocrinology.

[38] Sorensen SD, Conn PJ (2003). G protein-coupled receptor kinases regulate metabotropic glutamate receptor 5 function and expression. Neuropharmacology.

[39] Meng A, Ameroso D, Rios M (2023). mGluR5 in astrocytes in the ventromedial hypothalamus regulates pituitary adenylate cyclase-activating polypeptide neurons and glucose homeostasis. J Neurosci.

[40] Kammermeier PJ, Worley PF (2007). Homer 1a uncouples metabotropic glutamate receptor 5 from postsynaptic effectors. Proc Natl Acad Sci USA.

[41] Ye J, Yan X, Zhang W (2023). Integrative proteomic and phosphoproteomic analysis in the female goat hypothalamus to study the onset of puberty. BMC Genomics.

[42] Vastagh C, Rodolosse A, Solymosi N, Liposits Z (2016). Altered expression of genes encoding neurotransmitter receptors in GnRH neurons of proestrous mice. Front Cell Neurosci.

[43] McRee RC, Meyer DC (1993). GABA control of LHRH release is dependent on the steroid milieu. Neurosci Lett.

[44] Leonhardt, Shahab, Luft, Wuttke, Jarry (1999). Reduction of luteinzing hormone secretion induced by long-term feed restriction in male rats is associated with increased expression of GABA-synthesizing enzymes without alterations of GnRH gene expression. J Neuroendocrinol.

[45] Verma P, Shakya M (2021). Transcriptomics and sequencing analysis of gene expression profiling for major depressive disorder. Indian J Psychiatry.

[46] Dias MM, Cánovas A, Mantilla-Rojas C (2017). SNP detection using RNA-sequences of candidate genes associated with puberty in cattle. Genet Mol Res.

[47] Xiao L, Chen J, He X, Zhang X, Luo W (2024). Whole-transcriptome sequencing revealed the ceRNA regulatory network during the proliferation and differentiation of goose myoblast. Poult Sci.

[48] Garcia-Galiano D, Cara AL, Tata Z (2020). ERα signaling in GHRH/Kiss1 dual-phenotype neurons plays sex-specific roles in growth and puberty. J Neurosci.

[49] Smith JT, Cunningham MJ, Rissman EF, Clifton DK, Steiner RA (2005). Regulation of Kiss1 gene expression in the brain of the female mouse. Endocrinology.

[50] Mayer C, Acosta-Martinez M, Dubois SL (2010). Timing and completion of puberty in female mice depend on estrogen receptor α-signaling in kisspeptin neurons. Proc Natl Acad Sci USA.

[51] Wang X, Escobar JB, Mendelowitz D (2021). Sex differences in the hypothalamic oxytocin pathway to locus coeruleus and augmented attention with chemogenetic activation of hypothalamic oxytocin neurons. Int J Mol Sci.

[52] Semple E, Shalabi F, Hill JW (2019). Oxytocin neurons enable melanocortin regulation of male sexual function in mice. Mol Neurobiol.

[53] Bilbao MG, Garrigos D, Martinez-Morga M (2022). Prosomeric hypothalamic distribution of tyrosine hydroxylase positive cells in adolescent rats. Front Neuroanat.

[54] Cosenza G, Iannaccone M, Pico BA, Gallo D, Capparelli R, Pauciullo A (2017). Molecular characterisation, genetic variability and detection of a functional polymorphism influencing the promoter activity of OXT gene in goat and sheep. J Dairy Res.

[55] Parent AS, Rasier G, Matagne V (2008). Oxytocin facilitates female sexual maturation through a glia-to-neuron signaling pathway. Endocrinology.

