# Supplementary material for: Analysis of circular RNA (circRNA) characteristics and identification of key circRNAs in the hypothalamus during sexual maturation in female goats
Source: Anim Biosci. 2025 Jun 24;38(12):2545–57. doi: 10.5713/ab.25.0275 (PMC12580788; doi:10.5713/ab.25.0275)
Supplement: Supplementary file 1 [file ab-25-0275-Supplementary-1.pdf]

Supplement 1. Primers for circRNA validation and qPCR

Primer name	Nucleotide sequences (5’→3’)	Purpose
novel_circ_0002274-DF	CAATTCTCCCATGAAACTTCCAGT	Validation of the back-splicing junction sequence
novel_circ_0002274-DR	TTCTCCTTACTAATGCGGTTCTCAG	
novel_circ_0025302-DF	AGCTCAGAATGTCTGCATCCC	Validation of the back-splicing junction sequence
novel_circ_0025302-DR	TCCAAGCAATGAAATCAGCATCTG	
novel_circ_0005937-DF	CAACCTCGTCTCCTTGGATGT	Validation of the back-splicing junction sequence
novel_circ_0005937-DR	AAGCCCACTGATGATGTCTGTAA	
novel_circ_0006250-DF	CCCTCCAGTTCTTAAAGATCCGAA	Validation of the back-splicing junction sequence
novel_circ_0006250-DR	CCATTTACGAGGGCTGTCCAA	
novel_circ_0030183-DF	TGGAACTGTATCTATGGATGACTC	Validation of the back-splicing junction sequence
novel_circ_0030183-DR	CGTGTATCCTCAGCAAGATTCAG	
novel_circ_0002274-F	TCCCATGAAACTTCCAGTGCAT	Quantitative verification of sequencing results
novel_circ_0002274-R	TAGTCCCCAGTCATCACAGC	
novel_circ_0006250-F	GGAGTTTAAGTAGACCCACATTCT	Quantitative verification of sequencing results
novel_circ_0006250-R	GAGGGTAAGTAAGATGCTGCTCA	
novel_circ_0025302-F	GAATGTCTGCATCCCAGAGGA	Quantitative verification of sequencing results
novel_circ_0025302-R	GTAAAACTTTCTAGAGCAGCAGCAT	
novel_circ_0005937-F	ATTCCTCACAGGCGAAGGAC	Quantitative verification of sequencing results
novel_circ_0005937-R	TTTGCGAATGCAAGCTCGTT	
novel_circ_0030183-F	GGTTTTCCATCAAGCCACAAG	Quantitative verification of sequencing results
novel_circ_0030183-R	GCTCTGTACGTGTATCCTCAGC	
GAPDH-F	CTTATGACCACTGTCCACGC	Quantitative verification of sequencing results
GAPDH-F	CCGTTGAGCTCAGGGATGAC	
