# Supplementary material for: Analysis of circular RNA (circRNA) characteristics and identification of key circRNAs in the hypothalamus during sexual maturation in female goats
Source: Anim Biosci. 2025 Jun 24;38(12):2545–57. doi: 10.5713/ab.25.0275 (PMC12580788; doi:10.5713/ab.25.0275)
Supplement: Supplementary file 2 [file ab-25-0275-Supplementary-2.pdf]

Supplement 2. Summary of reads mapped to the goat genome

sample	clean_reads	clean_bases	Q20	Q30	GC_pct	total_map	unique_map	multi_map
HY_D1_1	85848850	12.88G	97.55	93.49	45.14	81399700(9[illegible]	76870758(89.[illegible]	4528942(5.28%)
HY_D1_2	87569092	13.14G	97.65	93.46	43.34	84054200(9[illegible]	79551494(90.[illegible]	4502706(5.14%)
HY_D1_3	91619800	13.74G	97.67	93.54	44.36	87920588(9[illegible]	83350417(90.[illegible]	4570171(4.99%)
HY_D1_4	88406732	13.26G	97.55	93.2	43.74	85035654(9[illegible]	81314352(91.[illegible]	3721302(4.21%)
HY_D1_5	92049850	13.81G	97.52	93.14	44.04	88262379(9[illegible]	84338640(91.[illegible]	3923739(4.26%)
HY_M2_1	82350226	12.35G	97.33	92.9	45.59	71164421(8[illegible]	65926844(80.[illegible]	5237577(6.36%)
HY_M2_2	83156586	12.47G	97.56	93.42	44.94	76234675(9[illegible]	71227120(85.[illegible]	5007555(6.02%)
HY_M2_3	92056562	13.81G	96.17	90.75	52.56	79972538(8[illegible]	69131693(75.[illegible]	10840845(11.78%)
HY_M2_4	85978180	12.9G	97.66	93.56	44.71	79022206(9[illegible]	74452180(86.[illegible]	4570026(5.32%)
HY_M2_5	87719830	13.16G	97.62	93.47	44.22	82130236(9[illegible]	77750240(88.[illegible]	4379996(4.99%)
HY_M4_1	88791728	13.32G	96.29	90.4	42.87	84439424(9[illegible]	80469771(90.[illegible]	3969653(4.47%)
HY_M4_2	88027246	13.2G	97.37	93.11	45.38	80685488(9[illegible]	75845189(86.[illegible]	4840299(5.5%)
HY_M4_3	91522488	13.73G	96.35	90.62	44.36	80611670(8[illegible]	76902087(84.[illegible]	3709583(4.05%)
HY_M4_4	84728830	12.71G	96.8	91.44	44.41	77034762(9[illegible]	73507217(86.[illegible]	3527545(4.16%)
HY_M4_5	90872690	13.63G	96.9	91.76	44.49	84838060(9[illegible]	80115259(88.[illegible]	4722801(5.2%)
HY_M6_1	93663688	14.05G	96.58	91.01	43.62	87623228(9[illegible]	83323573(88.[illegible]	4299655(4.59%)
HY_M6_2	83750452	12.56G	96.58	91.12	44.19	75898694(9[illegible]	71539551(85.[illegible]	4359143(5.2%)
HY_M6_3	85341082	12.8G	96.87	91.73	45.89	73431991(8[illegible]	69672646(81.[illegible]	3759345(4.41%)
HY_M6_4	94275920	14.14G	96.48	90.84	43.38	88597131(9[illegible]	83868300(88.[illegible]	4728831(5.02%)
HY_M6_5	86327080	12.95G	96.33	90.59	48.42	82032249(9[illegible]	74771239(86.[illegible]	7261010(8.41%)
