# Supplementary material for: Analysis of circular RNA (circRNA) characteristics and identification of key circRNAs in the hypothalamus during sexual maturation in female goats
Source: Anim Biosci. 2025 Jun 24;38(12):2545–57. doi: 10.5713/ab.25.0275 (PMC12580788; doi:10.5713/ab.25.0275)
Supplement: Supplementary file 5 [file ab-25-0275-Supplementary-5.pdf]

Supplement 5. Different expression patterns of circRNA were identified by mfuzz

gene id	D1	M2	M4	M6	membershi	Cluster
novel circ 0019665	5734.471	3399.592	3319.44	4359.159	0.716223	1
novel circ 0042265	1578.681	1101.004	837.4971	1061.208	0.507765	1
novel circ 0006462	1179.534	815.0735	1006.518	582.1394	0.33597	1
novel circ 0022094	1034.329	731.2909	437.1131	496.3277	0.403869	1
novel circ 0004724	912.4744	575.866	682.77	474.4846	0.415229	1
novel circ 0017476	611.6358	518.5203	317.6097	379.7046	0.34274	1
novel circ 0001762	755.7149	298.3271	357.9117	285.362	0.552831	1
novel circ 0039302	501.8127	281.346	386.2662	459.8013	0.437076	1
novel circ 0021778	552.5083	270.2513	398.6459	311.8748	0.495192	1
novel circ 0015594	624.7022	314.2514	160.4381	429.0133	0.517683	1
novel circ 0021775	547.55	261.6833	199.3553	251.755	0.570115	1
novel circ 0029385	430.8777	274.0946	284.5586	216.0848	0.446071	1
novel circ 0026782	619.4443	242.5717	150.3932	189.6765	0.535535	1
novel circ 0023626	606.6539	331.4661	24.33534	102.5783	0.394923	1
novel circ 0026345	323.0567	160.4365	164.6536	291.6483	0.479603	1
novel circ 0022058	302.4396	209.7119	154.7102	237.4377	0.500836	1
novel circ 0022179	283.1281	123.0241	230.2643	246.1983	0.386385	1
novel circ 0030347	364.7458	89.2917	212.3413	182.5421	0.569776	1
novel circ 0012948	272.0531	201.6879	132.2728	214.4699	0.436777	1
novel circ 0027542	353.3017	0	255.4303	188.7898	0.396469	1
novel circ 0037300	322.6488	100.2475	197.0861	119.8635	0.484531	1
novel circ 0003139	259.4073	93.40506	146.1005	178.8208	0.697345	1
novel circ 0035422	270.0491	164.7025	102.0841	100.6763	0.431384	1
novel circ 0013222	207.5693	50.94757	165.7399	165.925	0.369818	1
novel circ 0026899	223.844	124.2212	150.3782	79.17051	0.397553	1
novel circ 0012026	225.5584	151.1469	71.71242	116.317	0.419989	1
novel circ 0035608	211.2285	22.17348	105.0796	141.0062	0.562255	1
novel circ 0023079	163.4666	30.56827	132.3581	117.5483	0.366891	1
novel circ 0033102	147.5514	33.81212	128.2181	127.7788	0.318686	1
novel circ 0023630	206.1744	86.01897	34.06181	92.79964	0.547085	1
novel circ 0040161	173.0403	103.7384	84.25962	37.46739	0.38132	1
novel circ 0014228	151.3743	33.40152	82.64974	108.5132	0.570804	1
novel circ 0038327	163.4609	25.86142	105.3809	77.77207	0.479765	1
novel circ 0024484	168.9921	62.27827	94.67486	33.98804	0.4263	1
novel circ 0012126	203.7347	20.3887	39.11128	78.64684	0.767258	1
novel circ 0017598	225.5363	47.71491	18.07983	50.43081	0.593013	1
novel circ 0022605	131.9763	21.34631	73.86995	112.514	0.428127	1
novel circ 0021500	170.6107	0	79.44132	71.2659	0.573775	1
novel circ 0008850	148.0381	33.81212	7.132763	130.7166	0.429262	1
novel circ 0017206	135.7359	71.56208	21.19867	84.64273	0.467563	1
novel circ 0003201	109.5432	71.71186	11.195	110.4522	0.3331	1
novel circ 0028184	158.4676	0	58.05381	84.79189	0.653959	1
novel circ 0010150	175.0434	39.24376	35.2463	46.55054	0.618553	1
novel circ 0043162	176.0391	0	0	119.8447	0.564352	1
novel circ 0042224	105.2886	18.74397	88.91773	80.12404	0.341495	1
novel circ 0014261	118.6683	52.14548	9.42163	107.9178	0.394644	1
novel circ 0004192	139.5813	67.73913	57.35833	16.73033	0.403518	1
novel circ 0008264	141.2394	70.91458	15.12375	46.06737	0.450438	1
novel circ 0004882	155.2509	65.32462	8.124467	44.37606	0.478749	1
novel circ 0022516	114.8386	38.01929	17.17491	96.22968	0.460365	1
novel circ 0034968	104.0501	9.583306	83.04174	64.51538	0.36608	1
novel circ 0038729	149.0547	73.59206	15.6365	22.73707	0.419441	1
novel circ 0042264	96.41883	64.76642	13.34076	81.09836	0.362186	1
novel circ 0017449	137.5575	20.71714	36.15967	57.23003	0.763079	1
novel circ 0017652	127.7418	20.71714	29.23149	70.55304	0.756958	1
novel circ 0012313	126.5575	16.18391	86.25597	17.72554	0.382066	1
novel circ 0039910	163.257	8.738013	53.04998	21.19982	0.552639	1
novel circ 0042863	138.5916	29.37186	12.05322	57.95768	0.660492	1
novel circ 0023183	108.5951	20.0542	34.82879	65.53386	0.750714	1
novel circ 0030250	102.0463	41.69271	12.66352	68.3573	0.508603	1
novel circ 0020102	123.5377	26.28406	18.29796	53.36239	0.69752	1
novel circ 0009989	123.266	20.3887	0	74.33639	0.582201	1
novel circ 0014029	82.13691	55.8693	9.592993	68.4656	0.357293	1
novel circ 0025612	77.5443	7.281677	60.7482	67.34101	0.332582	1
novel circ 0001817	96.09537	11.15776	45.34145	59.60215	0.61354	1
novel circ 0037138	125.7817	0	63.21113	22.47816	0.478858	1
novel circ 0043894	73.45657	52.81651	7.743063	71.53015	0.326085	1
novel circ 0031426	85.05753	63.87303	9.840919	43.5927	0.341278	1
novel circ 0035011	91.04905	0	58.44997	52.07959	0.442671	1
novel circ 0039067	86.27161	0	35.33111	73.71363	0.423626	1
novel circ 0016663	79.5343	10.19435	32.73679	70.38874	0.428545	1
novel circ 0023358	96.22575	0	46.99229	47.29532	0.559893	1
novel circ 0037674	133.1973	0	22.75237	34.34663	0.717835	1
novel circ 0021945	92.72253	0	33.01587	63.9353	0.564286	1
novel circ 0029800	81.10889	33.54157	0	71.66386	0.396517	1
novel circ 0030591	99.8156	51.17099	5.416311	28.92093	0.430015	1
novel circ 0036663	89.69271	0	29.90478	64.78484	0.544802	1
novel circ 0040104	75.00314	5.825342	34.32939	68.17104	0.390701	1
novel circ 0006048	87.03565	55.94091	26.31875	10.08541	0.357024	1
novel circ 0019263	68.11065	0	57.26044	53.68689	0.320916	1
novel circ 0010949	65.52713	52.0658	5.530759	54.60844	0.317262	1
novel circ 0015470	100.097	8.738013	32.9757	34.73846	0.685373	1
novel circ 0040789	76.51044	0	32.34536	65.43748	0.420219	1
novel circ 0014714	72.42939	8.385393	57.08905	36.20059	0.373765	1
novel circ 0000638	59.66886	44.87349	2.708156	63.88663	0.305187	1
novel circ 0004767	77.64696	4.791653	20.54006	63.40981	0.490023	1
novel circ 0020632	68.91322	7.281677	53.97866	33.55184	0.374829	1
novel circ 0018218	127.1589	0	36.5601	0	0.515078	1
novel circ 0023660	82.37566	42.79643	5.416311	32.30673	0.441687	1
novel circ 0036728	78.02597	0	48.04728	34.4898	0.46117	1
novel circ 0014148	71.53252	31.42834	5.416311	50.23208	0.464663	1
novel circ 0037303	96.35431	8.385393	23.61099	29.68563	0.705954	1
novel circ 0002670	56.26096	41.51741	5.416311	45.70204	0.337904	1
novel circ 0036328	85.01951	0	63.51842	0	0.342521	1
novel circ 0015039	80.9344	3.59374	12.16767	51.51063	0.647321	1
novel circ 0015988	86.29704	0	50.84589	8.955809	0.416288	1
novel circ 0016254	59.76604	45.10896	0	40.91008	0.32848	1
novel circ 0012695	86.06689	0	14.97106	43.35391	0.757838	1
novel circ 0011852	69.0057	32.62625	4.062233	36.077	0.471849	1
novel circ 0039375	63.61652	11.08072	3.318456	62.04594	0.385298	1
novel circ 0036257	69.09648	34.16835	0	32.66269	0.443491	1
novel circ 0004224	92.38285	0	14.89486	28.22642	0.752601	1
novel circ 0031712	53.71829	37.12533	0	42.30918	0.34397	1
novel circ 0002255	79.37655	4.791653	32.03129	15.90618	0.545979	1
novel circ 0016388	69.924	0	33.08953	28.50609	0.565609	1
novel circ 0008599	66.55555	0	46.16373	17.81266	0.393704	1
novel circ 0037875	130.2037	0	0	0	0.590007	1
novel circ 0008618	64.95014	0	15.83927	48.46565	0.539102	1
novel circ 0012481	65.99328	0	6.770389	55.92913	0.456818	1
novel circ 0023575	59.03924	29.29093	2.708156	36.36128	0.447055	1
novel circ 0027246	77.88333	0	17.60301	29.52676	0.761194	1
novel circ 0017906	74.26805	0	25.31782	24.31255	0.652195	1
novel circ 0034564	88.74689	19.94188	0	13.99715	0.54692	1
novel circ 0004404	81.86342	11.65068	0	29.09066	0.656039	1
novel circ 0027985	85.33822	0	8.849215	27.69985	0.770324	1
novel circ 0017423	48.89112	18.93236	0	50.04479	0.35693	1
novel circ 0023781	53.55814	25.15618	0	36.65752	0.433707	1
novel circ 0008794	60.58645	5.825342	5.530759	42.40904	0.572186	1
novel circ 0013943	47.97538	34.62724	0	31.74574	0.340637	1
novel circ 0014297	64.35197	0	18.80458	31.18998	0.723987	1
novel circ 0004560	61.20182	0	34.32939	18.66172	0.477174	1
novel circ 0024220	66.24071	0	46.87399	0	0.355192	1
novel circ 0029691	50.65555	4.369006	33.29882	23.73839	0.452922	1
novel circ 0015052	75.03139	0	5.530759	31.43034	0.772679	1
novel circ 0020338	48.69575	0	40.19364	22.49964	0.342203	1
novel circ 0024963	61.34772	0	6.770389	43.05961	0.572833	1
novel circ 0002233	57.51235	21.91506	0	30.99523	0.499474	1

novel	circ	0029574	71.87447	5.825342	7.743063	23.78047	0.736326	1
novel	circ	0020339	65.56009	16.01969	3.318456	23.71046	0.609486	1
novel	circ	0024211	63.19814	0	23.85852	20.47357	0.620659	1
novel	circ	0023167	49.59414	0	17.36984	40.42256	0.465202	1
novel	circ	0022798	70.42882	29.71358	6.770389	0	0.415919	1
novel	circ	0012346	52.03692	34.04868	0	20.70489	0.365667	1
novel	circ	0015531	46.2502	0	19.43391	40.91264	0.400556	1
novel	circ	0033407	57.97052	0	35.2808	13.13839	0.431793	1
novel	circ	0039451	50.67888	0	18.19468	37.27842	0.526563	1
novel	circ	0032610	58.91482	11.65068	35.05352	0	0.383446	1
novel	circ	0001807	60.5178	7.281677	0	37.52935	0.587189	1
novel	circ	0025557	47.72699	0	31.06453	26.13191	0.438935	1
novel	circ	0015720	50.01254	0	38.77444	15.65206	0.358326	1
novel	circ	0036144	65.06155	0	13.83661	25.37103	0.771631	1
novel	circ	0012633	50.77186	0	28.11354	25.32494	0.50799	1
novel	circ	0038559	57.70365	31.9341	0	14.25126	0.397961	1
novel	circ	0006755	51.8098	8.738013	0	43.31686	0.451943	1
novel	circ	0026139	51.89438	0	11.195	39.75659	0.522882	1
novel	circ	0017015	51.17215	17.54605	0	33.32617	0.492353	1
novel	circ	0012857	74.82317	16.01969	0	9.759362	0.543243	1
novel	circ	0035637	50.58843	0	12.91145	36.99899	0.551481	1
novel	circ	0013935	56.69297	0	28.27866	14.87832	0.507099	1
novel	circ	0032743	49.2281	0	11.44292	38.99279	0.499711	1
novel	circ	0004055	50.01493	0	13.54078	35.399	0.571037	1
novel	circ	0039156	63.82599	0	15.25723	19.83465	0.718752	1
novel	circ	0014725	57.61726	22.16143	18.95709	0	0.412412	1
novel	circ	0026534	47.29663	11.65068	0	39.65572	0.441573	1
novel	circ	0020997	57.80469	0	27.67322	12.14876	0.503013	1
novel	circ	0004142	65.12553	0	27.29182	5.200208	0.489498	1
novel	circ	0015648	45.14383	0	23.96374	28.31203	0.50333	1
novel	circ	0033973	54.71409	0	8.317253	33.67293	0.659979	1
novel	circ	0012253	42.35623	0	17.76537	35.82243	0.427607	1
novel	circ	0015596	49.02445	0	19.30043	27.27287	0.625529	1
novel	circ	0019776	44.27797	22.31551	0	28.81595	0.425801	1
novel	circ	0001964	62.15602	0	13.90315	19.11974	0.726029	1
novel	circ	0026578	64.72129	14.46916	0	15.40039	0.573857	1
novel	circ	0020797	51.49452	3.59374	0	38.81314	0.509187	1
novel	circ	0015558	49.70586	31.91379	0	11.17416	0.359161	1
novel	circ	0039404	49.67252	0	5.530759	37.24906	0.53207	1
novel	circ	0020626	53.69485	0	8.849215	29.85058	0.716704	1
novel	circ	0031889	45.16349	0	8.849215	38.28404	0.456382	1
novel	circ	0004412	61.12779	0	0	30.92756	0.691479	1
novel	circ	0030205	56.63004	0	6.770389	28.39234	0.750245	1
novel	circ	0023950	51.38444	9.583306	0	29.90933	0.584081	1
novel	circ	0032018	37.31476	0	21.19867	32.29396	0.379397	1
novel	circ	0027621	75.09404	0	0	15.35674	0.698906	1
novel	circ	0010129	47.92225	0	14.0176	27.94266	0.673057	1
novel	circ	0012430	41.52213	22.31551	0	25.2034	0.416719	1
novel	circ	0040947	51.13577	0	6.636911	31.15171	0.661533	1
novel	circ	0012023	56.31618	0	0	31.98341	0.648808	1
novel	circ	0004128	42.03624	0	20.07799	24.9144	0.550629	1
novel	circ	0004657	53.72695	0	6.770389	26.5309	0.757177	1
novel	circ	0015815	49.61289	5.825342	0	31.569	0.578076	1
novel	circ	0037724	61.45168	11.97913	13.54078	0	0.487191	1
novel	circ	0016430	39.7066	0	10.83262	36.32336	0.40089	1
novel	circ	0037612	54.42668	0	19.96141	12.31424	0.582372	1
novel	circ	0039981	47.78829	0	6.770389	30.88303	0.629033	1
novel	circ	0017419	49.16474	13.69389	0	22.5782	0.56699	1
novel	circ	0022088	48.34035	16.62107	0	20.38408	0.527547	1
novel	circ	0023765	44.09616	29.34891	0	11.83945	0.353279	1
novel	circ	0021729	44.23012	0	23.992	16.64067	0.505061	1
novel	circ	0012761	54.30502	0	14.13244	16.33135	0.698777	1
novel	circ	0025555	52.63082	0	7.743063	24.26111	0.779257	1
novel	circ	0020573	47.84659	0	14.89486	21.43631	0.713335	1
novel	circ	0019108	36.67292	13.10702	0	34.01127	0.390763	1
novel	circ	0034977	37.32824	0	15.98198	30.4314	0.446875	1
novel	circ	0033288	54.9465	14.56335	0	13.96255	0.552489	1
novel	circ	0022898	39.04011	17.47603	0	26.88495	0.441253	1
novel	circ	0036901	56.8358	17.9687	0	8.365167	0.494405	1
novel	circ	0032729	44.72195	0	26.2043	11.7442	0.452151	1
novel	circ	0011442	46.61152	12.67954	0	23.15006	0.566409	1
novel	circ	0031869	55.45031	0	8.124467	18.45058	0.771194	1
novel	circ	0005197	42.70006	29.10938	0	10.07528	0.344954	1
novel	circ	0010128	41.54859	0	14.89486	25.35999	0.620993	1
novel	circ	0039885	42.88691	0	5.416311	33.44123	0.509061	1
novel	circ	0033742	46.848	0	4.424607	30.45276	0.615488	1
novel	circ	0010610	40.76623	0	20.01595	20.82325	0.556769	1
novel	circ	0003446	54.04784	0	8.124467	19.21977	0.780417	1
novel	circ	0042845	32.58148	0	23.22919	25.49605	0.366446	1
novel	circ	0026514	45.34289	0	0	35.88946	0.486678	1
novel	circ	0000596	45.53762	0	13.27382	20.52054	0.728942	1
novel	circ	0006022	60.0371	0	0	19.03109	0.740822	1
novel	circ	0023773	47.40326	0	27.03427	4.477904	0.422789	1
novel	circ	0035097	37.29371	0	28.28313	12.95711	0.369193	1
novel	circ	0043877	45.80052	0	32.40329	0	0.355239	1
novel	circ	0039262	37.29844	0	18.80458	22.00816	0.53249	1
novel	circ	0029957	44.6292	0	13.65523	19.81404	0.717532	1
novel	circ	0026958	40.48382	0	0	37.47643	0.406889	1
novel	circ	0017381	37.8018	0	10.94707	28.96849	0.516108	1
novel	circ	0027030	37.93156	0	10.45122	29.23325	0.514258	1
novel	circ	0015085	42.58399	0	7.743063	27.07852	0.643791	1
novel	circ	0030371	30.68137	0	24.57406	22.053	0.344415	1
novel	circ	0033405	44.63454	0	16.59228	15.99961	0.639466	1
novel	circ	0013026	52.66797	0	18.90017	5.59738	0.529929	1
novel	circ	0041953	35.12858	16.77079	0	24.81016	0.426418	1
novel	circ	0036003	46.11244	0	19.92976	10.55928	0.53972	1
novel	circ	0016713	46.37005	0	10.83262	19.03109	0.765052	1
novel	circ	0026829	35.95961	0	6.770389	33.39944	0.398984	1
novel	circ	0011713	55.47831	20.3887	0	0	0.43522	1
novel	circ	0038626	37.51315	17.12341	21.19867	0	0.357261	1
novel	circ	0035151	45.70527	15.57287	0	14.40151	0.518848	1
novel	circ	0036363	42.74302	0	14.76138	17.89102	0.680663	1
novel	circ	0012318	45.31564	0	19.91073	9.759362	0.529656	1
novel	circ	0026283	38.02391	0	9.478545	27.13554	0.568311	1
novel	circ	0029931	39.32386	7.281677	0	27.7349	0.521	1
novel	circ	0042418	36.11931	0	14.87583	23.09552	0.572502	1
novel	circ	0007766	48.40713	0	20.90244	4.160166	0.485504	1
novel	circ	0006877	50.27165	0	0	22.89775	0.719664	1
novel	circ	0015264	56.59859	0	9.478545	6.716857	0.623822	1
novel	circ	0019097	60.21915	0	0	12.56835	0.701013	1
novel	circ	0021554	43.73441	19.39125	9.478545	0	0.404883	1
novel	circ	0027126	31.98362	0	22.38999	17.78054	0.408898	1
novel	circ	0042030	45.69301	0	4.424607	21.89999	0.756548	1
novel	circ	0015771	36.61479	0	8.124467	26.7323	0.554973	1
novel	circ	0027151	41.94468	0	29.36792	0	0.357653	1
novel	circ	0038532	51.25872	0	7.389135	12.4805	0.716578	1
novel	circ	0020287	45.19505	0	5.530759	20.10937	0.781163	1
novel	circ	0012471	70.65721	0	0	0	0.590007	1
novel	circ	0044085	36.7731	0	7.743063	25.99546	0.576716	1
novel	circ	0019066	49.25179	0	21.15036	0	0.45857	1
novel	circ	0013832	49.29748	0	10.83262	10.0911	0.658881	1
novel	circ	0021894	51.30318	10.78122	8.124467	0	0.491493	1
novel	circ	0038411	55.95723	7.281677	0	6.24025	0.581132	1
novel	circ	0003962	42.30955	9.583306	0	17.03965	0.602147	1
novel	circ	0026342	37.28287	8.738013	0	22.82188	0.551097	1
novel	circ	0022028	42.93035	0	0	25.42542	0.630811	1
novel	circ	0009584	50.41701	13.52967	0	4.182584	0.499834	1
novel	circ	0027029	34.41701	0	9.478545	23.18466	0.601405	1
novel	circ	0003973	46.6351	0	17.83191	2.238952	0.494824	1

novel circ 0000412	48.88048	0	6.770389	10.40042	0.696734	1
novel circ 0008696	61.03789	0	0	4.182584	0.625466	1
novel circ 0009292	45.10603	0	5.416311	14.22885	0.76587	1
novel circ 0036150	44.42811	0	5.416311	13.83828	0.763684	1
novel circ 0026823	55.37734	0	0	7.836333	0.665384	1
novel circ 0041555	44.32587	0	0	18.67215	0.733479	1
novel circ 0037943	37.07291	0	0	25.04885	0.568145	1
novel circ 0012170	41.0722	0	0	19.00389	0.716147	1
novel circ 0020282	39.83902	7.281677	12.66352	0	0.468691	1
novel circ 0034845	35.82756	0	20.17769	3.358428	0.425827	1
novel circ 0014212	51.47949	0	6.636911	0	0.567292	1
novel circ 0011294	46.97503	0	0	10.87702	0.711952	1
novel circ 0043310	44.50156	0	2.708156	10.60412	0.723306	1
novel circ 0009177	39.53317	0	9.478545	8.401134	0.653717	1
novel circ 0001367	44.31649	0	11.8053	0	0.523045	1
novel circ 0025648	31.69727	0	2.708156	18.17973	0.680516	1
novel circ 0043445	34.24076	0	4.062233	12.57604	0.785293	1
novel circ 0031891	40.51818	0	9.955367	0	0.530622	1
novel circ 0021811	5937.465	5123.416	11103.86	7243.94	0.675008	2
novel circ 0010238	663.0447	645.0263	1242.472	1120.161	0.436993	2
novel circ 0014613	710.1975	642.1324	1199.594	517.3857	0.574888	2
novel circ 0022202	374.3272	486.4361	1030.605	1043.138	0.357302	2
novel circ 0031169	557.2659	498.6085	978.3738	860.6021	0.469635	2
novel circ 0001710	710.0497	478.0908	993.684	516.7229	0.686243	2
novel circ 0037466	389.5422	783.4727	807.426	506.555	0.309905	2
novel circ 0002866	449.6614	545.697	929.0826	534.0258	0.535503	2
novel circ 0041270	510.0182	343.9378	779.8314	536.8858	0.792555	2
novel circ 0026881	243.4356	473.4387	900.2666	526.2448	0.461302	2
novel circ 0043464	648.879	370.4452	679.5535	392.2434	0.46204	2
novel circ 0000293	507.908	314.0861	609.7636	368.111	0.625755	2
novel circ 0016112	304.4155	266.9105	642.6436	212.2189	0.598152	2
novel circ 0020756	323.5615	217.2787	441.7547	440.3881	0.38285	2
novel circ 0015924	196.1039	426.2904	476.0384	307.5369	0.331516	2
novel circ 0025165	245.1215	212.0614	603.821	294.2846	0.67	2
novel circ 0021810	364.4983	186.4125	540.9345	227.6269	0.688985	2
novel circ 0005937	488.4644	0	488.9687	248.0724	0.429213	2
novel circ 0014500	287.5305	336.7405	407.0879	134.9801	0.355254	2
novel circ 0003004	188.6148	321.7959	424.3229	221.9548	0.398934	2
novel circ 0008356	178.6432	207.3006	514.7037	231.811	0.58719	2
novel circ 0006278	288.7828	165.2756	374.8239	289.0473	0.64758	2
novel circ 0038824	197.3873	129.2516	569.0154	175.997	0.693726	2
novel circ 0042300	228.7748	316.6036	401.2804	112.9812	0.368589	2
novel circ 0011377	167.9781	168.5541	391.9971	324.2617	0.466795	2
novel circ 0032370	155.5091	236.005	374.7077	260.6041	0.449152	2
novel circ 0001803	234.3815	216.7378	483.7255	86.41126	0.506048	2
novel circ 0000039	291.8635	144.4361	344.6055	214.023	0.607991	2
novel circ 0007038	236.5395	111.2744	352.586	248.238	0.688641	2
novel circ 0035731	144.4745	219.7851	391.1341	192.4648	0.491638	2
novel circ 0002606	212.8721	91.03143	329.3194	303.9603	0.433026	2
novel circ 0029017	256.9962	93.55011	328.516	239.4815	0.575296	2
novel circ 0025972	168.2713	145.479	423.0357	178.2847	0.664078	2
novel circ 0026865	131.4115	114.8584	377.2914	171.6277	0.657401	2
novel circ 0042498	94.64437	205.7272	311.5386	155.7401	0.416485	2
novel circ 0006455	178.045	126.8421	345.3363	111.5769	0.636984	2
novel circ 0004390	93.62033	94.03491	297.4135	241.5797	0.456443	2
novel circ 0006099	82.50952	200.8958	252.2995	190.4421	0.346502	2
novel circ 0006582	148.4091	76.52639	299.1866	189.3357	0.706321	2
novel circ 0025993	245.0622	56.33339	246.2251	159.4926	0.427251	2
novel circ 0014288	182.9712	82.82275	268.0682	107.381	0.677366	2
novel circ 0041030	186.0638	66.24636	236.5586	140.1834	0.638526	2
novel circ 0007853	95.9843	56.2259	288.6284	157.9698	0.660331	2
novel circ 0008774	85.63877	155.1661	244.0635	112.682	0.440603	2
novel circ 0035724	47.8807	130.0581	298.9026	114.5959	0.482534	2
novel circ 0012435	116.2732	62.44181	194.4777	201.4571	0.360686	2
novel circ 0012461	191.3193	34.91222	195.7483	151.2624	0.399267	2
novel circ 0040081	27.29826	178.6497	187.3064	176.281	0.285309	2
novel circ 0019399	48.96827	193.9146	219.6348	94.0318	0.328207	2
novel circ 0031269	102.708	32.01521	195.8591	206.7557	0.3545	2
novel circ 0020788	132.6414	55.78998	207.361	135.2654	0.730614	2
novel circ 0020926	77.14912	157.761	235.2846	56.96644	0.401948	2
novel circ 0023262	33.36289	0	482.2935	0	0.635236	2
novel circ 0034905	115.8278	106.3132	208.0202	54.87872	0.501822	2
novel circ 0005310	148.1712	121.0429	159.4776	53.16591	0.349484	2
novel circ 0022222	124.0468	45.42848	173.5952	136.9017	0.544156	2
novel circ 0027227	56.48215	83.99429	270.6147	57.25804	0.553379	2
novel circ 0023150	86.62489	30.80886	191.0191	145.3336	0.541751	2
novel circ 0020543	51.21093	13.10702	250.9116	137.9969	0.604092	2
novel circ 0017223	115.8155	48.87508	139.8464	137.1851	0.367765	2
novel circ 0007917	31.7979	111.2842	162.3322	125.0505	0.356665	2
novel circ 0021389	85.56945	31.75679	157.7565	151.5236	0.404508	2
novel circ 0023919	117.8435	14.72758	204.9416	81.95796	0.787498	2
novel circ 0028041	136.3813	32.00677	154.8262	88.92222	0.521299	2
novel circ 0020071	126.4677	39.44877	137.1476	100.0212	0.46575	2
novel circ 0001114	151.3292	18.93236	146.2557	65.61894	0.418538	2
novel circ 0031021	49.16967	5.825342	181.1333	141.1971	0.485048	2
novel circ 0023258	28.25415	104.6977	149.5775	89.69909	0.372041	2
novel circ 0031326	128.7841	31.59256	118.1173	86.40622	0.359015	2
novel circ 0010287	105.5182	13.10702	158.7096	85.65928	0.679967	2
novel circ 0024647	64.54272	27.33032	175.146	80.57345	0.758078	2
novel circ 0038200	51.8857	10.19435	226.02	51.46693	0.732373	2
novel circ 0030276	82.34471	20.81134	125.3231	106.7879	0.471312	2
novel circ 0009906	49.32462	22.3377	173.8518	89.46977	0.66193	2
novel circ 0034518	86.59863	27.05933	141.6488	79.05303	0.780908	2
novel circ 0026482	70.44285	34.41103	157.2064	68.36603	0.804533	2
novel circ 0010910	49.51818	25.00647	128.6035	116.7705	0.426802	2
novel circ 0029883	114.2111	19.26081	112.1848	73.8644	0.407334	2
novel circ 0035006	72.46401	21.13979	154.5635	66.96661	0.848318	2
novel circ 0003383	52.18686	29.82107	166.5555	49.732	0.707643	2
novel circ 0003784	62.95316	7.281677	147.2222	80.4532	0.724464	2
novel circ 0028509	102.0556	17.54605	93.40402	84.57565	0.315146	2
novel circ 0017371	50.29838	17.47603	148.756	80.81974	0.682629	2
novel circ 0002780	22.98051	106.72	135.9124	19.83465	0.349735	2
novel circ 0017765	79.43133	7.187479	123.4794	68.48035	0.674853	2
novel circ 0024617	60.82376	33.20468	153.6255	30.86163	0.660317	2
novel circ 0015606	90.86027	14.37496	101.4337	67.53847	0.474901	2
novel circ 0036323	54.93773	7.281677	152.0314	56.16352	0.822844	2
novel circ 0015100	49.88333	0	187.7046	30.23506	0.755968	2
novel circ 0036778	63.29335	8.385393	113.5879	77.58165	0.605818	2
novel circ 0007134	35.56266	10.78122	143.0669	65.43688	0.684314	2
novel circ 0026484	63.32481	19.77254	102.3276	63.96055	0.715671	2
novel circ 0009161	44.51665	10.78122	116.4494	76.92851	0.602796	2
novel circ 0007137	58.94888	16.18391	125.5735	46.49756	0.839175	2
novel circ 0009455	61.96711	12.33175	93.782	76.33364	0.493204	2
novel circ 0031311	71.89415	11.65068	99.73034	61.03061	0.615076	2
novel circ 0028398	64.14202	11.97913	113.1022	50.45278	0.809806	2
novel circ 0002907	82.29754	11.65068	80.3312	64.03459	0.368241	2
novel circ 0025947	39.50759	18.48554	116.6075	60.67392	0.693558	2
novel circ 0013910	39.90132	13.10702	133.9485	47.85473	0.754946	2
novel circ 0011104	9.100661	0	198.7712	26.52448	0.648412	2
novel circ 0028405	71.79913	5.825342	88.14938	66.05455	0.467243	2
novel circ 0021656	73.06778	10.19435	91.95001	50.47166	0.575817	2
novel circ 0004552	41.1358	0	96.36088	82.92039	0.452969	2
novel circ 0007016	72.76796	32.30951	105.9075	8.365167	0.49102	2
novel circ 0010494	36.74127	0	113.8239	65.79717	0.644063	2
novel circ 0016448	48.82653	8.310537	99.6544	52.86058	0.768987	2
novel circ 0003051	57.39481	13.17705	104.8285	33.78878	0.774116	2
novel circ 0013839	68.87746	42.18907	89.46194	6.970973	0.419374	2
novel circ 0030541	44.0427	17.12341	80.21595	65.54571	0.514056	2
novel circ 0017010	30.54675	0	166.6109	8.955809	0.685249	2

novel circ 0018895	73.35554	11.97913	103.9666	11.13114	0.546108	2
novel circ 0034408	77.35081	5.825342	81.76729	34.0701	0.467563	2
novel circ 0014828	70.64202	42.53801	78.61129	5.200208	0.37449	2
novel circ 0022755	44.21746	0	110.3804	41.36548	0.843936	2
novel circ 0023080	32.58083	5.989566	108.672	47.61519	0.740454	2
novel circ 0019257	53.70165	0	73.28308	66.08418	0.401414	2
novel circ 0025046	63.33624	10.19435	79.37603	39.48158	0.584844	2
novel circ 0014120	69.9288	0	86.54684	34.25878	0.554397	2
novel circ 0005964	43.31443	7.281677	79.11026	59.53551	0.547593	2
novel circ 0038914	48.97905	8.385393	70.19652	59.80414	0.454913	2
novel circ 0015485	65.19167	36.50917	81.72072	2.788389	0.411133	2
novel circ 0016246	94.90211	7.281677	74.67445	7.836333	0.341044	2
novel circ 0037682	63.25184	0	86.32279	32.87627	0.614075	2
novel circ 0034474	69.25174	0	62.33633	50.00333	0.333168	2
novel circ 0005918	49.98978	5.825342	70.13358	54.32374	0.496304	2
novel circ 0026861	57.28634	0	75.32823	45.11999	0.54841	2
novel circ 0004323	30.57633	11.13384	96.43466	39.53235	0.749672	2
novel circ 0040073	38.14326	5.989566	130.4325	0	0.647385	2
novel circ 0043934	44.30447	0	70.85698	57.18932	0.476222	2
novel circ 0021753	64.97117	7.281677	91.82923	6.970973	0.541209	2
novel circ 0026876	51.15228	0	83.90638	35.5328	0.724602	2
novel circ 0029796	54.81511	0	78.99867	36.32965	0.64316	2
novel circ 0002274	61.63472	4.791653	75.21738	25.42155	0.546808	2
novel circ 0028352	45.295	4.791653	59.32216	56.18128	0.379769	2
novel circ 0036027	56.58911	8.310537	66.77321	32.57831	0.541162	2
novel circ 0002976	41.18505	26.3167	92.29246	4.182584	0.538177	2
novel circ 0008241	27.20533	7.281677	67.94343	58.09271	0.459736	2
novel circ 0019289	26.82046	5.825342	68.83053	56.62228	0.478466	2
novel circ 0037185	25.09006	0	67.77167	62.71213	0.416289	2
novel circ 0030972	22.8757	16.01969	108.7903	6.970973	0.609222	2
novel circ 0002077	30.36277	0	72.51074	48.56677	0.59076	2
novel circ 0007264	69.71396	4.369006	56.93944	20.1607	0.347498	2
novel circ 0036164	52.38261	0	52.81737	45.89974	0.350671	2
novel circ 0001057	62.68338	0	65.95801	21.52416	0.462333	2
novel circ 0001842	30.29977	15.92549	96.35558	7.280291	0.618736	2
novel circ 0036344	48.48569	2.912671	54.09016	43.17342	0.409915	2
novel circ 0018619	62.05368	0	57.52583	28.6356	0.393463	2
novel circ 0020865	16.79139	0	78.7057	52.36414	0.539822	2
novel circ 0030993	50.7479	7.281677	85.79059	2.238952	0.572257	2
novel circ 0024856	53.78032	0	79.84454	12.31424	0.60223	2
novel circ 0009585	59.06347	0	51.37923	33.18383	0.347934	2
novel circ 0008254	32.84491	4.369006	72.85508	32.6693	0.826601	2
novel circ 0028240	46.98842	0	50.82805	44.1755	0.369141	2
novel circ 0026704	15.64574	0	91.9441	32.98885	0.697159	2
novel circ 0028795	38.32258	0	64.05692	36.83527	0.655744	2
novel circ 0034793	8.553014	0	66.57651	63.57203	0.391963	2
novel circ 0038861	35.69403	7.281677	54.49413	39.40447	0.572458	2
novel circ 0020917	9.099361	0	72.56014	55.13423	0.467858	2
novel circ 0033191	13.46836	0	104.3002	16.31553	0.692378	2
novel circ 0035046	31.87587	0	62.27006	38.4827	0.642758	2
novel circ 0023554	9.259278	0	72.7289	50.21355	0.501104	2
novel circ 0012358	32.90621	4.369006	64.36883	29.48306	0.819598	2
novel circ 0034279	33.39443	28.39887	68.77632	0	0.464561	2
novel circ 0013745	34.7969	25.10495	69.86119	0	0.484173	2
novel circ 0026830	46.6974	0	48.22807	33.88921	0.406554	2
novel circ 0000209	39.2553	0	73.08896	15.60062	0.729088	2
novel circ 0028587	42.69633	7.281677	71.96698	5.576778	0.590806	2
novel circ 0013061	28.60325	0	82.24776	16.54723	0.795872	2
novel circ 0036937	39.44408	0	69.57704	16.49682	0.719534	2
novel circ 0012081	50.87979	8.385393	65.90586	0	0.48225	2
novel circ 0015907	38.05214	0	57.11088	29.88933	0.645138	2
novel circ 0015897	19.27109	0	80.97495	24.31813	0.76512	2
novel circ 0040192	29.14911	0	66.03189	28.66951	0.814588	2
novel circ 0003206	33.42562	0	74.4002	14.583	0.771687	2
novel circ 0042788	20.92583	0	50.47397	49.74856	0.388305	2
novel circ 0006467	38.99164	20.3887	58.16664	2.238952	0.46817	2
novel circ 0030171	28.3709	0	64.71076	26.42244	0.831663	2
novel circ 0032805	33.06448	0	43.37862	42.97487	0.354775	2
novel circ 0023814	38.5337	0	64.51347	16.33135	0.70223	2
novel circ 0023263	2.873047	4.369006	85.4891	26.48638	0.605375	2
novel circ 0018762	59.04654	0	53.63594	6.421536	0.392799	2
novel circ 0030923	11.98815	0	77.72845	29.07917	0.679789	2
novel circ 0012295	26.14339	0	52.63517	39.84735	0.524481	2
novel circ 0033617	14.88691	19.39125	83.6358	0	0.539693	2
novel circ 0001191	52.18357	19.16661	43.40526	2.788389	0.329769	2
novel circ 0002441	27.69355	0	66.21206	22.36613	0.853008	2
novel circ 0027225	19.43686	0	87.58429	8.824734	0.71545	2
novel circ 0032286	33.28015	0	55.21056	26.82622	0.709672	2
novel circ 0007429	25.19969	0	70.11794	19.80563	0.836339	2
novel circ 0004468	17.76184	0	73.54159	23.46779	0.763974	2
novel circ 0013015	39.39242	0	41.34708	33.23934	0.382553	2
novel circ 0021062	36.14519	2.912671	63.41422	9.779964	0.661168	2
novel circ 0023818	39.69102	0	61.60514	10.62472	0.629365	2
novel circ 0034708	36.3407	0	42.49213	32.32914	0.433048	2
novel circ 0022841	25.64312	0	46.2399	38.38114	0.469855	2
novel circ 0005351	28.13322	0	56.09045	24.54243	0.799073	2
novel circ 0012836	28.88935	0	43.68678	36.03901	0.458271	2
novel circ 0039054	9.751341	0	54.71471	43.90229	0.457721	2
novel circ 0032814	22.39025	0	78.91022	6.970973	0.721504	2
novel circ 0021231	23.74082	0	46.2395	38.05079	0.476967	2
novel circ 0044164	26.30985	0	52.64423	28.79785	0.710951	2
novel circ 0010921	15.16464	0	78.59575	13.77519	0.729014	2
novel circ 0014416	19.18139	0	81.94942	5.576778	0.703306	2
novel circ 0016131	35.22914	0	62.09022	7.81573	0.657278	2
novel circ 0013549	31.22666	0	44.87938	28.33934	0.569175	2
novel circ 0024329	31.10176	0	47.63457	25.70675	0.646669	2
novel circ 0005067	21.04515	36.31419	46.72573	0	0.349203	2
novel circ 0037931	43.91496	22.31551	37.66828	0	0.318912	2
novel circ 0024323	26.44201	0	60.89646	16.49926	0.828408	2
novel circ 0042256	10.53588	0	59.7658	33.1757	0.593897	2
novel circ 0014590	0	4.369006	96.03736	2.788389	0.595906	2
novel circ 0028828	28.84829	0	59.12068	14.87832	0.788163	2
novel circ 0005471	30.16785	0	46.24851	26.04048	0.634102	2
novel circ 0001671	2.806919	0	83.58555	15.63146	0.64291	2
novel circ 0004196	40.01804	26.95791	33.26901	0	0.291719	2
novel circ 0032430	22.82949	0	58.22077	19.02062	0.851099	2
novel circ 0013570	27.91194	33.76592	38.15602	0	0.315292	2
novel circ 0014789	36.66429	0	35.92649	26.46408	0.377478	2
novel circ 0009780	9.513452	0	46.51609	43.0111	0.407948	2
novel circ 0018631	27.83554	0	65.08004	5.576778	0.701494	2
novel circ 0003135	17.02397	0	63.3184	17.91162	0.788878	2
novel circ 0001227	17.72908	0	68.77349	11.46287	0.75564	2
novel circ 0013432	46.18648	0	47.15551	4.182584	0.433108	2
novel circ 0043610	26.6879	7.281677	63.10482	0	0.599716	2
novel circ 0015559	19.78846	0	39.21729	37.9317	0.394069	2
novel circ 0004952	26.18074	0	51.78325	18.91015	0.81782	2
novel circ 0015443	18.4183	0	62.05992	16.2015	0.804341	2
novel circ 0004350	33.01454	0	34.74867	28.53355	0.377853	2
novel circ 0005241	35.9492	11.08072	49.06491	0	0.474481	2
novel circ 0038867	32.84337	0	38.10541	25.04928	0.469345	2
novel circ 0013777	18.77126	0	76.73848	0	0.670864	2
novel circ 0020695	8.010494	0	57.69245	29.80148	0.598326	2
novel circ 0023025	44.24238	0	44.94001	6.24025	0.435311	2
novel circ 0029875	4.210379	0	82.63161	8.16283	0.647562	2
novel circ 0005199	28.52907	0	58.00493	8.320333	0.712462	2
novel circ 0014588	21.75459	0	36.5632	36.28184	0.375366	2
novel circ 0006151	21.24096	0	59.47346	12.85707	0.805625	2
novel circ 0011680	16.56481	0	38.07715	38.11162	0.381084	2
novel circ 0036597	21.41973	0	38.07715	33.18476	0.443009	2

novel circ 0041780	18.06647	0	52.63934	21.67461	0.795944	2
novel circ 0002650	8.681401	0	49.8444	33.56783	0.523158	2
novel circ 0024215	12.13386	7.281677	72.3925	0	0.605092	2
novel circ 0013595	18.63421	0	67.47916	5.59738	0.717644	2
novel circ 0006874	40.52085	0	34.36672	15.37734	0.35913	2
novel circ 0024896	18.77013	0	64.33455	6.716857	0.73159	2
novel circ 0003801	16.32531	13.10702	59.91669	0	0.556086	2
novel circ 0034781	15.1828	0	48.02195	25.37268	0.683963	2
novel circ 0036360	35.37526	0	44.85405	8.320333	0.538689	2
novel circ 0020336	16.43125	0	59.35887	11.7442	0.775727	2
novel circ 0003056	13.43813	8.310537	65.06828	0	0.596785	2
novel circ 0028094	12.82952	0	61.01029	12.21391	0.743578	2
novel circ 0003141	43.37349	0	37.78487	4.160166	0.378303	2
novel circ 0035049	2.806919	7.281677	74.11399	0	0.576674	2
novel circ 0016757	5.746094	0	55.35	22.9002	0.630558	2
novel circ 0010539	32.38915	19.77765	31.81207	0	0.335542	2
novel circ 0002762	22.4513	0	44.10416	16.37437	0.815276	2
novel circ 0022771	20.17472	0	31.38744	31.14222	0.370435	2
novel circ 0015216	5.267825	26.53044	48.51937	0	0.407328	2
novel circ 0039154	17.46199	0	55.26896	6.478553	0.741193	2
novel circ 0000441	21.73762	0	38.47565	18.94171	0.731569	2
novel circ 0036849	8.461825	0	62.35207	8.320333	0.691771	2
novel circ 0039849	17.09671	0	45.16714	14.25787	0.847605	2
novel circ 0024273	30.14581	0	31.05149	14.90074	0.446525	2
novel circ 0000892	12.15892	0	51.59283	11.15356	0.761034	2
novel circ 0007650	22.69645	0	43.54075	7.81573	0.718706	2
novel circ 0042693	15.426	0	49.16221	6.970973	0.756177	2
novel circ 0036269	18.53307	0	38.85859	12.74122	0.831757	2
novel circ 0011108	24.13492	9.583306	36.04522	0	0.482483	2
novel circ 0008845	32.02983	8.738013	27.15562	0	0.343736	2
novel circ 0040663	30.40427	7.281677	29.79546	0	0.387446	2
novel circ 0009549	6.160186	0	47.07644	13.09537	0.692104	2
novel circ 0032981	36.1327	0	27.61631	0	0.33946	2
novel circ 0037332	11.35993	2.395826	49.70045	0	0.64282	2
novel circ 0011397	15.03197	0	47.24843	0	0.670132	2
novel circ 0026878	17.72158	0	40.88074	0	0.646304	2
novel circ 0038645	865.6376	1132.632	457.9797	699.3745	0.711968	3
novel circ 0027507	585.9139	1172.013	498.0401	553.1085	0.742929	3
novel circ 0021835	366.0989	860.9801	837.6263	728.2968	0.313777	3
novel circ 0038527	837.7637	882.835	400.3713	642.1721	0.50295	3
novel circ 0034145	430.2753	1157.015	628.7259	504.4923	0.553517	3
novel circ 0038982	639.2214	689.8464	366.2102	784.8569	0.338826	3
novel circ 0025302	793.6557	720.3279	377.8818	444.2635	0.384545	3
novel circ 0023964	518.5764	787.5865	381.6716	607.3409	0.715101	3
novel circ 0044578	706.4745	714.5556	394.8962	296.128	0.415019	3
novel circ 0009702	412.8582	906.7647	354.8479	380.4157	0.723299	3
novel circ 0039588	302.2127	744.4697	331.7201	473.6453	0.633165	3
novel circ 0022247	556.5608	686.1618	232.1402	360.3732	0.615276	3
novel circ 0005231	282.6057	509.3237	236.1265	607.2693	0.356678	3
novel circ 0018548	198.5032	524.3196	481.9728	396.9158	0.336896	3
novel circ 0000753	299.8698	507.0979	393.7485	222.9727	0.426726	3
novel circ 0028097	206.7268	502.6286	494.8986	167.3667	0.324495	3
novel circ 0042974	426.3211	566.9764	99.3906	268.6791	0.637355	3
novel circ 0021772	341.372	506.1736	275.3289	163.2441	0.522571	3
novel circ 0002576	231.8881	493.4384	206.6424	344.5978	0.659223	3
novel circ 0004091	119.3809	574.1269	256.8463	281.3014	0.542604	3
novel circ 0028250	304.1145	349.3696	178.409	399.1636	0.349475	3
novel circ 0013987	158.5823	469.6706	196.1222	277.242	0.615025	3
novel circ 0038987	124.5632	608.2224	152.3349	203.1731	0.669731	3
novel circ 0005606	294.8642	409.6939	177.598	196.157	0.679262	3
novel circ 0038172	71.55331	447.0589	244.5374	287.8717	0.452696	3
novel circ 0012987	158.4203	402.7113	161.5102	271.9486	0.622065	3
novel circ 0043595	238.3841	442.7172	108.7752	157.6781	0.778311	3
novel circ 0009648	280.8732	277.1331	141.262	183.8406	0.448235	3
novel circ 0044040	101.335	311.1786	255.6229	208.8479	0.375188	3
novel circ 0029294	113.3818	414.0998	180.9001	164.1481	0.583451	3
novel circ 0042215	214.5285	441.8414	100.192	102.035	0.706926	3
novel circ 0036797	185.6479	391.2309	110.3475	168.6006	0.829597	3
novel circ 0004394	109.9516	357.9971	155.7115	230.6214	0.564512	3
novel circ 0002788	117.9374	369.1768	214.7831	110.4437	0.487824	3
novel circ 0043336	112.335	336.573	174.0294	170.1266	0.56054	3
novel circ 0039105	97.07635	297.9628	168.026	200.7311	0.499196	3
novel circ 0010685	94.87264	379.7168	112.1736	154.2941	0.667713	3
novel circ 0018090	179.4007	336.4911	71.32842	113.0464	0.77654	3
novel circ 0042270	60.15271	310.1114	192.553	122.1348	0.449903	3
novel circ 0029976	80.81837	327.4185	136.0492	137.631	0.585633	3
novel circ 0014978	56.50789	305.2996	129.0908	171.3953	0.531798	3
novel circ 0036619	172.3183	340.4675	9.955367	133.5946	0.801204	3
novel circ 0038643	100.6902	381.7472	48.23254	115.1322	0.784	3
novel circ 0020115	143.8797	254.7984	54.03167	191.8551	0.625747	3
novel circ 0020651	72.5628	295.1278	142.074	125.6278	0.542886	3
novel circ 0014069	215.0588	181.5764	57.28867	157.0662	0.341316	3
novel circ 0039204	55.63901	208.236	178.5059	145.9433	0.35269	3
novel circ 0021774	187.6715	213.1037	59.17025	122.7175	0.547125	3
novel circ 0039889	8.619142	266.4436	63.21113	232.3249	0.442395	3
novel circ 0018445	98.88532	338.7261	46.02023	64.33114	0.747443	3
novel circ 0042943	106.5347	291.6822	61.86973	84.78107	0.767055	3
novel circ 0033030	53.47079	198.0522	150.0507	139.7183	0.387768	3
novel circ 0037405	123.7441	232.1057	68.08574	114.5365	0.859909	3
novel circ 0013210	93.71634	262.2987	73.20842	102.8233	0.753325	3
novel circ 0012123	94.26197	181.7521	46.70398	206.7922	0.394372	3
novel circ 0014944	87.15375	262.3387	95.2086	79.22055	0.643306	3
novel circ 0008018	98.42323	165.2833	38.86782	218.9658	0.33842	3
novel circ 0016427	133.9565	186.8344	43.31208	111.2041	0.681789	3
novel circ 0009843	81.68711	236.088	65.12375	88.86834	0.744946	3
novel circ 0002691	121.1582	210.4311	32.00224	103.9407	0.790991	3
novel circ 0018053	90.91977	193.9888	57.79159	124.3625	0.732556	3
novel circ 0009523	53.27213	172.3781	136.7007	99.26213	0.390462	3
novel circ 0028786	149.9518	182.8565	19.91073	106.9719	0.560093	3
novel circ 0001644	124.3807	214.146	27.26316	92.16787	0.78001	3
novel circ 0024463	119.3808	170.4249	45.992	112.1692	0.688457	3
novel circ 0010597	160.0217	135.7425	43.62658	107.9464	0.352257	3
novel circ 0029966	100.7765	194.0739	44.53751	107.3065	0.826119	3
novel circ 0021982	109.2898	200.5103	41.55734	90.74984	0.839488	3
novel circ 0001229	0	332.9173	68.30682	34.30659	0.587446	3
novel circ 0002521	116.4122	195.5013	33.35395	87.1986	0.784137	3
novel circ 0027193	37.993	271.1079	33.82391	68.82311	0.707522	3
novel circ 0013510	41.84311	171.1934	65.45382	131.8298	0.499441	3
novel circ 0027461	48.79823	208.8204	30.49543	114.9135	0.670416	3
novel circ 0001413	64.03395	190.1171	25.64191	108.1426	0.718899	3
novel circ 0003068	53.86082	163.649	82.36648	85.82939	0.566798	3
novel circ 0004596	60.79641	201.3631	38.59164	84.328	0.768687	3
novel circ 0031587	90.76396	188.4946	21.89414	77.50978	0.847863	3
novel circ 0028684	123.085	134.8116	30.06947	82.44312	0.512877	3
novel circ 0007750	82.0126	129.804	19.68184	138.094	0.421867	3
novel circ 0021260	87.75546	151.547	25.75636	103.4564	0.671424	3
novel circ 0006250	93.41116	153.6505	13.54078	99.59972	0.6532	3
novel circ 0002834	36.48641	149.6591	60.6024	97.38755	0.540796	3
novel circ 0019988	71.437	204.8967	25.78679	39.10962	0.759989	3
novel circ 0009344	108.4026	112.757	111.5047	6.24025	0.29111	3
novel circ 0034245	63.00947	147.2696	20.25425	107.3724	0.617391	3
novel circ 0041517	68.09472	165.957	60.75335	36.53651	0.617673	3
novel circ 0009172	33.45222	191.6634	13.65523	72.28202	0.739702	3
novel circ 0010757	28.24768	234.2718	38.39202	9.736945	0.63021	3
novel circ 0015049	81.44639	138.0511	18.48049	63.48464	0.775205	3
novel circ 0005530	57.33915	201.2381	21.19867	21.48114	0.719551	3
novel circ 0007013	17.30054	185.4472	52.71173	42.19665	0.58908	3
novel circ 0043420	40.89983	151.5984	80.93325	21.49514	0.477819	3
novel circ 0008758	108.5358	119.3983	54.66944	7.836333	0.403531	3

novel circ 0022926	38.91826	127.4317	15.6196	103.5024	0.535304	3
novel circ 0015940	57.65706	176.3551	16.72576	29.56499	0.763457	3
novel circ 0012161	56.49002	145.3812	20.15906	51.20318	0.8472	3
novel circ 0008507	8.010494	88.69392	91.0932	79.36228	0.293044	3
novel circ 0027294	67.02355	114.4945	74.73069	10.42283	0.411106	3
novel circ 0043721	42.05162	143.2482	0	80.26588	0.699307	3
novel circ 0008113	34.34985	206.0894	9.955367	5.576778	0.701161	3
novel circ 0041076	57.50808	112.3129	0	85.49185	0.572659	3
novel circ 0041210	47.40863	125.4885	67.01625	9.759362	0.468651	3
novel circ 0040957	17.3678	182.8097	0	39.74212	0.749911	3
novel circ 0011283	65.0672	126.7801	9.42163	29.21083	0.745935	3
novel circ 0027442	29.64296	144.6048	10.40619	44.22561	0.776989	3
novel circ 0044356	84.7176	92.94896	0	51.02073	0.495933	3
novel circ 0028465	60.17897	121.6075	2.708156	43.9011	0.80732	3
novel circ 0021897	58.97981	106.5701	52.48694	10.07528	0.479809	3
novel circ 0031886	69.08976	87.23254	0	69.74685	0.487013	3
novel circ 0027359	42.13985	120.3463	5.416311	57.2666	0.792706	3
novel circ 0022513	75.37374	78.52717	6.636911	62.46468	0.436959	3
novel circ 0040330	64.10699	104.9011	4.062233	49.78657	0.712177	3
novel circ 0000622	48.11742	98.46206	13.15937	60.70908	0.723968	3
novel circ 0020063	4.471022	149.8296	30.40063	33.9928	0.608284	3
novel circ 0019414	10.53565	143.1049	18.27084	46.71191	0.662608	3
novel circ 0009962	25.39583	133.847	8.317253	44.78825	0.766512	3
novel circ 0022946	59.03252	78.73995	5.416311	57.76009	0.541065	3
novel circ 0031387	59.49961	116.5336	3.318456	15.08202	0.696869	3
novel circ 0036460	30.01334	110.6244	0	44.08653	0.807513	3
novel circ 0040358	26.69852	116.1836	36.23623	5.576778	0.565999	3
novel circ 0025456	34.92263	80.68955	0	62.4345	0.567881	3
novel circ 0024464	20.30209	138.8779	0	16.2221	0.759861	3
novel circ 0011620	56.61331	79.01166	2.708156	36.1866	0.632312	3
novel circ 0037567	7.017298	73.72851	5.416311	85.29887	0.391472	3
novel circ 0000550	30.3494	99.70586	7.380689	32.60722	0.835973	3
novel circ 0003818	41.84573	77.46235	0	48.40911	0.655912	3
novel circ 0032694	37.69754	89.05657	2.708156	35.2545	0.843657	3
novel circ 0035177	35.58799	97.21373	0	31.69288	0.860772	3
novel circ 0002889	36.92467	81.5341	0	44.88975	0.727156	3
novel circ 0031669	58.52119	54.85771	4.062233	43.2089	0.399451	3
novel circ 0007584	42.58677	65.23562	5.416311	42.72826	0.627692	3
novel circ 0021527	17.55318	101.6568	0	35.42158	0.772718	3
novel circ 0021721	49.12554	64.89649	0	39.55882	0.566169	3
novel circ 0013107	27.24151	70.25707	0	55.95826	0.552478	3
novel circ 0028866	43.32698	63.06322	3.318456	42.89899	0.588281	3
novel circ 0007713	26.11646	94.0778	0	30.99808	0.841932	3
novel circ 0042685	36.90711	61.50661	0	48.35869	0.54536	3
novel circ 0000914	39.76339	71.56174	0	34.25089	0.731164	3
novel circ 0020072	28.4076	96.40203	0	19.11974	0.829176	3
novel circ 0001063	52.61521	58.04258	3.318456	29.55361	0.507444	3
novel circ 0037045	18.67094	109.9858	0	14.13448	0.771903	3
novel circ 0043043	52.68275	68.99039	0	20.86808	0.585213	3
novel circ 0016011	25.63124	87.35944	26.58143	0	0.556939	3
novel circ 0044373	55.75275	60.06811	2.708156	20.10937	0.493496	3
novel circ 0013982	5.04243	85.74264	8.849215	38.89862	0.62938	3
novel circ 0004877	33.35867	76.91687	26.7318	0	0.527644	3
novel circ 0008642	26.21596	81.22668	2.708156	23.86192	0.853561	3
novel circ 0041367	19.46993	63.28185	47.84667	2.788389	0.379877	3
novel circ 0016721	43.28373	49.61025	0	40.06112	0.457907	3
novel circ 0038603	49.80771	59.97059	0	19.88705	0.546405	3
novel circ 0010849	43.81097	55.76998	0	28.72502	0.570625	3
novel circ 0003998	56.49618	53.12	0	17.91162	0.434531	3
novel circ 0028195	39.22302	40.20247	0	47.29767	0.345425	3
novel circ 0006859	15.6987	57.70274	0	53.16525	0.481211	3
novel circ 0044212	16.3693	61.58269	48.59429	0	0.367406	3
novel circ 0025298	30.47754	62.64063	0	31.59141	0.751828	3
novel circ 0010736	50.72735	47.87067	2.708156	23.36594	0.433367	3
novel circ 0001372	37.81956	49.17969	36.31279	0	0.354495	3
novel circ 0028318	0	81.36861	18.07983	23.41879	0.584663	3
novel circ 0029257	26.96978	61.39307	30.76239	0	0.461927	3
novel circ 0001015	50.54263	40.66029	27.74939	0	0.305664	3
novel circ 0023516	25.56632	63.18512	0	29.93018	0.789354	3
novel circ 0026577	9.125721	93.51323	0	15.90618	0.749616	3
novel circ 0024095	33.59508	41.83767	0	41.30918	0.421134	3
novel circ 0006921	27.54272	55.44097	0	33.42358	0.681302	3
novel circ 0011849	25.98324	65.95808	0	21.90813	0.85486	3
novel circ 0028202	10.08486	88.157	15.37168	0	0.610738	3
novel circ 0036069	4.210379	78.41571	30.29818	0	0.49797	3
novel circ 0022756	37.03306	35.49256	0	40.34676	0.342161	3
novel circ 0040811	42.97197	45.86309	20.40659	2.788389	0.398616	3
novel circ 0007005	38.77953	38.282	34.82524	0	0.298938	3
novel circ 0039354	38.89563	52.27471	0	20.6588	0.606316	3
novel circ 0036056	18.99771	57.32983	0	35.25817	0.667646	3
novel circ 0021137	0	78.74922	10.83262	21.49514	0.626032	3
novel circ 0043784	5.108558	95.0648	10.83262	0	0.631339	3
novel circ 0027253	42.3688	36.26271	0	31.0549	0.360152	3
novel circ 0016654	25.64288	62.19412	0	21.0265	0.848231	3
novel circ 0002294	20.43036	55.10715	0	32.53348	0.693986	3
novel circ 0037110	20.20577	82.39763	5.416311	0	0.683793	3
novel circ 0024239	48.61316	48.47813	10.69914	0	0.411156	3
novel circ 0038721	32.63625	32.71562	0	41.31773	0.32687	3
novel circ 0000847	42.22278	42.12265	0	21.24944	0.456671	3
novel circ 0038761	19.89484	61.46549	0	23.90696	0.835084	3
novel circ 0018240	4.210379	75.09692	0	24.79474	0.705299	3
novel circ 0023692	19.15116	70.53344	14.13244	0	0.61101	3
novel circ 0017202	45.40282	42.66967	0	15.03719	0.43447	3
novel circ 0031766	14.26604	68.88363	19.76862	0	0.562313	3
novel circ 0033864	30.58482	60.57368	7.380689	4.182584	0.639147	3
novel circ 0022874	26.64306	58.12661	0	17.19671	0.816422	3
novel circ 0018268	18.23823	71.67079	11.77704	0	0.630314	3
novel circ 0005425	32.59545	51.86839	0	17.03784	0.688615	3
novel circ 0037517	24.89442	47.58808	0	28.66706	0.675481	3
novel circ 0026987	29.47572	43.57169	0	26.06829	0.612904	3
novel circ 0017890	25.60287	49.83337	0	23.48179	0.758387	3
novel circ 0017942	10.21842	70.82164	0	14.95775	0.777976	3
novel circ 0017471	19.2586	60.22324	0	16.2015	0.856518	3
novel circ 0031141	16.85445	43.32584	0	35.43567	0.540312	3
novel circ 0000028	41.88033	33.39186	0	20.24533	0.361796	3
novel circ 0023379	34.68166	42.21128	0	18.20753	0.554976	3
novel circ 0012199	26.53306	38.42742	0	29.62019	0.530594	3
novel circ 0010215	12.6376	34.24681	0	47.442	0.349027	3
novel circ 0020006	36.62606	38.0338	0	19.57393	0.473954	3
novel circ 0029393	13.50142	56.81071	0	22.72412	0.788195	3
novel circ 0036686	26.97908	49.66997	0	15.97719	0.75741	3
novel circ 0036955	27.449	48.63545	0	15.79571	0.740507	3
novel circ 0035871	34.68409	37.49477	0	18.45058	0.494074	3
novel circ 0021259	28.5822	50.27497	0	10.40042	0.699526	3
novel circ 0019126	33.71786	33.95461	0	20.46779	0.451962	3
novel circ 0002241	22.60917	57.49158	2.212304	0	0.671716	3
novel circ 0015004	0	56.0171	16.48785	9.382792	0.548837	3
novel circ 0027298	41.72418	39.28685	0	0	0.418853	3
novel circ 0015554	17.33272	45.19955	0	18.44045	0.833028	3
novel circ 0019422	34.08757	36.93852	8.849215	0	0.430787	3
novel circ 0031580	39.64263	35.08187	4.062233	0	0.390389	3
novel circ 0036197	16.53075	48.14117	13.15937	0	0.571016	3
novel circ 0010067	31.02602	31.11318	0	12.95711	0.462143	3
novel circ 0001600	31.9308	28.41303	0	12.84307	0.410921	3
novel circ 0001816	19.03321	45.75017	0	5.576778	0.750095	3
novel circ 0028356	510.7549	703.4911	995.0464	1099.385	0.325752	4
novel circ 0000191	90.91094	396.2309	707.2791	904.1892	0.331387	4
novel circ 0013180	244.5556	170.3404	729.1232	861.1802	0.367176	4
novel circ 0019197	410.3945	272.7102	496.9389	513.6094	0.369006	4

novel circ 0011310	190.2748	326.7747	498.382	590.7024	0.333446	4
novel circ 0035567	325.9668	377.0055	181.8524	613.1713	0.391347	4
novel circ 0030879	266.2475	347.3425	188.5356	457.9472	0.337069	4
novel circ 0000264	311.5105	217.5717	320.903	405.8436	0.621	4
novel circ 0039464	267.7627	128.5254	380.4537	425.534	0.401819	4
novel circ 0013146	189.943	157.1605	334.1504	440.3311	0.430446	4
novel circ 0024524	242.5005	175.2527	308.5448	391.9567	0.496499	4
novel circ 0015155	238.6423	134.3426	326.8429	388.705	0.442648	4
novel circ 0004072	186.9313	204.5174	247.3536	394.5145	0.467061	4
novel circ 0036989	173.7398	260.1171	114.5238	375.3595	0.343645	4
novel circ 0035561	193.6394	138.6471	201.9357	370.2316	0.616006	4
novel circ 0000474	220.6821	110.565	204.1864	273.1247	0.614232	4
novel circ 0006196	73.79939	119.5819	275.0749	335.2959	0.350239	4
novel circ 0018921	243.6867	99.43939	174.7034	274.4178	0.602206	4
novel circ 0043122	57.16818	73.72004	204.0186	285.7164	0.390881	4
novel circ 0024688	219.5646	89.74489	52.46577	239.6199	0.412105	4
novel circ 0018923	154.3237	112.7498	69.69136	259.8562	0.506863	4
novel circ 0007393	128.3169	75.89399	185.78	204.8291	0.391572	4
novel circ 0034060	172.1344	0	184.9179	233.1943	0.450358	4
novel circ 0009180	150.1331	155.3877	68.02981	199.2929	0.318402	4
novel circ 0034561	191.4405	45.04615	153.9874	177.4679	0.359763	4
novel circ 0020459	124.0409	60.22097	170.2461	192.2609	0.411805	4
novel circ 0002511	95.10002	74.0819	131.1928	238.2478	0.532301	4
novel circ 0039865	0	169.1032	132.8425	231.4283	0.293735	4
novel circ 0030935	125.2222	69.6813	129.7472	199.418	0.658884	4
novel circ 0039506	83.75283	158.4432	32.64119	238.6263	0.327837	4
novel circ 0043108	154.8825	114.2453	11.195	228.3801	0.382399	4
novel circ 0025219	116.7189	49.03237	133.7034	183.5321	0.553509	4
novel circ 0029855	124.7968	45.28645	121.3474	184.0535	0.642068	4
novel circ 0006284	135.7396	32.53205	132.7267	164.2679	0.464295	4
novel circ 0013185	67.95021	46.74279	135.4517	209.8545	0.464836	4
novel circ 0030939	64.75929	61.71892	110.1694	204.0573	0.487135	4
novel circ 0009811	92.76846	105.434	52.25649	185.2259	0.410552	4
novel circ 0030179	96.75426	40.48968	134.0866	139.632	0.362241	4
novel circ 0011358	104.4569	47.14009	89.95461	169.2365	0.763476	4
novel circ 0023543	120.4924	21.65664	122.9096	129.8194	0.363798	4
novel circ 0018049	95.10073	29.12671	122.2802	135.8414	0.4026	4
novel circ 0026445	107.0729	29.71358	102.1568	141.0989	0.552003	4
novel circ 0000034	99.15353	39.42213	79.79982	146.9641	0.786996	4
novel circ 0034683	100.7099	87.8847	24.14023	150.8665	0.375869	4
novel circ 0030576	111.7528	16.77079	92.74893	121.2899	0.448856	4
novel circ 0044361	19.32874	72.22871	85.79225	164.4079	0.375001	4
novel circ 0007875	77.13607	19.77765	117.8743	124.4307	0.364202	4
novel circ 0009787	98.4868	29.94783	69.74413	136.0116	0.763805	4
novel circ 0001127	143.2111	22.87389	29.0269	138.9773	0.39218	4
novel circ 0009236	110.8808	23.20717	86.45103	107.112	0.392634	4
novel circ 0041575	99.21244	29.65685	54.46919	141.4864	0.744775	4
novel circ 0017595	95.03121	9.583306	75.5978	134.5081	0.650949	4
novel circ 0028364	71.1593	5.989566	104.3895	120.7701	0.407719	4
novel circ 0028659	83.50558	9.583306	74.91643	125.5638	0.630757	4
novel circ 0011036	84.4033	26.54249	51.8447	122.3849	0.779456	4
novel circ 0031678	83.6381	16.86498	70.53713	112.8218	0.622878	4
novel circ 0039540	85.47364	14.72758	36.18287	145.0016	0.739748	4
novel circ 0035647	95.59485	2.912671	82.48662	99.46108	0.397855	4
novel circ 0003512	84.60926	12.07333	83.59684	99.06983	0.423483	4
novel circ 0026854	58.62747	16.62107	43.18215	157.9142	0.663662	4
novel circ 0043206	79.69557	7.281677	70.05232	111.2592	0.587868	4
novel circ 0005091	65.99887	27.14026	50.89282	120.8816	0.746511	4
novel circ 0005926	80.3667	11.65068	26.20216	141.6994	0.703878	4
novel circ 0009449	75.51378	20.40199	70.10414	92.92518	0.515019	4
novel circ 0039352	70.866	9.583306	73.36487	102.0224	0.524417	4
novel circ 0006128	54.34001	17.64025	51.23653	109.7947	0.703995	4
novel circ 0037408	77.76703	9.695627	67.67398	74.84843	0.3594	4
novel circ 0042898	37.43413	0	77.4089	113.0955	0.464104	4
novel circ 0011512	79.21033	32.9547	4.062233	109.1336	0.448451	4
novel circ 0024217	63.66877	0	75.32161	82.76871	0.384841	4
novel circ 0002651	47.40768	63.79874	0	109.4253	0.346025	4
novel circ 0028871	64.83903	12.33175	42.21826	100.1562	0.795472	4
novel circ 0040252	61.30004	8.221168	46.51609	101.2193	0.764813	4
novel circ 0006932	40.08779	14.56335	52.05751	106.5785	0.595123	4
novel circ 0002655	28.4448	7.281677	73.11582	104.3273	0.436043	4
novel circ 0019801	39.61692	44.83363	12.05322	112.0651	0.438618	4
novel circ 0002694	61.73136	5.989566	48.20629	87.80171	0.661581	4
novel circ 0002375	38.41889	0	73.7897	89.81628	0.410151	4
novel circ 0044099	25.32048	33.95461	6.770389	133.393	0.464834	4
novel circ 0015927	78.72566	28.343	7.628615	83.98664	0.382407	4
novel circ 0020257	40.43932	14.93649	51.31329	89.02421	0.588548	4
novel circ 0013928	47.01176	2.912671	43.45755	98.64046	0.710183	4
novel circ 0017083	42.94391	40.60229	12.91145	94.23568	0.443852	4
novel circ 0010281	47.37101	4.369006	41.00791	96.81346	0.733182	4
novel circ 0031087	27.79251	44.61223	4.062233	112.1051	0.410377	4
novel circ 0039023	51.4454	11.81491	53.6325	69.91945	0.501876	4
novel circ 0013158	67.413	27.31775	6.636911	83.79611	0.430148	4
novel circ 0000564	29.45946	0	37.64784	115.6785	0.613492	4
novel circ 0005958	2.235511	40.66632	52.73845	84.69347	0.337883	4
novel circ 0028854	63.08508	7.187479	45.57016	63.98355	0.435521	4
novel circ 0040473	47.62985	38.10866	8.849215	84.42141	0.427412	4
novel circ 0030354	58.20904	43.1201	7.066222	68.84203	0.330208	4
novel circ 0035648	42.09025	7.281677	0	125.6134	0.590336	4
novel circ 0044297	58.83321	8.385393	26.4332	81.33278	0.704221	4
novel circ 0030163	63.64971	25.08615	2.708156	81.17573	0.427904	4
novel circ 0003756	60.96155	11.97913	8.849215	90.61912	0.57175	4
novel circ 0026516	50.34893	7.281677	38.08794	76.68867	0.736589	4
novel circ 0021092	45.97741	5.540358	34.04259	86.72447	0.780208	4
novel circ 0003848	69.45467	5.825342	19.54836	76.90032	0.50677	4
novel circ 0034383	59.69498	4.369006	36.7421	69.38352	0.565508	4
novel circ 0035133	51.92193	0	47.08771	65.86422	0.490147	4
novel circ 0038443	36.19469	41.85694	0	86.49576	0.383796	4
novel circ 0002688	65.56317	17.22956	0	79.80291	0.434609	4
novel circ 0008678	7.824289	26.44829	28.39797	98.06653	0.437275	4
novel circ 0041116	45.25056	0	39.36367	70.71403	0.636449	4
novel circ 0039028	48.2533	7.281677	11.06152	88.61634	0.661561	4
novel circ 0018055	53.59657	0	44.95631	55.04729	0.397043	4
novel circ 0025730	22.21654	46.04484	2.212304	82.32789	0.349013	4
novel circ 0000656	50.35122	0	8.849215	90.47901	0.685925	4
novel circ 0010787	52.91996	6.271853	22.86682	65.55412	0.626466	4
novel circ 0004353	28.18221	0	19.92976	95.2957	0.663252	4
novel circ 0043627	16.85469	0	32.56348	93.75586	0.559936	4
novel circ 0017789	18.19784	42.59805	4.062233	77.6541	0.35249	4
novel circ 0007354	48.24451	0	24.61232	68.32046	0.7191	4
novel circ 0035331	52.26162	0	14.26553	74.01938	0.662151	4
novel circ 0006064	41.61054	0	25.06835	73.75883	0.80201	4
novel circ 0025203	38.52836	7.281677	8.124467	86.47606	0.640111	4
novel circ 0017162	45.77191	0	16.85923	76.67924	0.7583	4
novel circ 0000776	44.112	0	35.36292	59.10718	0.577275	4
novel circ 0005498	22.28396	0	8.849215	105.6904	0.603705	4
novel circ 0043985	35.56098	0	41.00363	57.7779	0.508653	4
novel circ 0040370	45.41207	0	2.708156	85.62169	0.646186	4
novel circ 0003114	6.00787	0	52.37666	74.62182	0.396413	4
novel circ 0016014	41.00936	4.369006	29.74933	56.41412	0.671895	4
novel circ 0036141	24.5968	0	31.04763	75.81476	0.625135	4
novel circ 0018115	32.53746	8.385393	17.22161	71.25224	0.701855	4
novel circ 0005702	50.68643	0	16.24893	59.83905	0.567917	4
novel circ 0040632	44.22876	0	35.4012	46.65355	0.423974	4
novel circ 0035184	36.45598	7.281677	14.19899	68.02553	0.709959	4
novel circ 0017303	28.51141	0	25.44149	71.5921	0.708676	4
novel circ 0034829	50.93773	0	9.478545	63.59637	0.564036	4
novel circ 0029476	53.21175	0	21.66524	49.12797	0.401707	4
novel circ 0020828	24.68412	0	28.89717	70.01465	0.642803	4

novel circ 0042575	40.65044	0	24.48785	58.20076	0.71177	4
novel circ 0034998	34.19226	0	15.48613	72.36038	0.76762	4
novel circ 0021291	33.47793	0	34.77579	53.7625	0.558635	4
novel circ 0036346	38.29865	4.791653	0	78.62354	0.598863	4
novel circ 0005922	13.88915	5.825342	24.10644	77.03924	0.546467	4
novel circ 0005458	38.73241	0	16.48785	65.43413	0.779645	4
novel circ 0003937	40.61939	25.33327	0	54.46406	0.375437	4
novel circ 0019754	38.81471	0	5.416311	76.12839	0.677742	4
novel circ 0022593	28.02677	0	28.92721	62.93914	0.672031	4
novel circ 0041357	48.8939	0	21.53177	49.33238	0.46452	4
novel circ 0028897	31.46568	3.59374	26.11305	58.38218	0.747202	4
novel circ 0030491	55.09981	7.281677	0	56.74834	0.394805	4
novel circ 0038646	53.62197	0	10.33677	54.48432	0.44303	4
novel circ 0043830	32.0861	0	0	86.23721	0.631485	4
novel circ 0013162	34.61129	0	22.12304	61.46259	0.795265	4
novel circ 0036535	48.04298	0	25.72748	44.25557	0.398498	4
novel circ 0026026	51.27795	0	18.93806	47.53376	0.402679	4
novel circ 0004400	51.60699	0	13.27382	52.25021	0.451184	4
novel circ 0038717	36.86059	3.59374	31.39588	45.23734	0.515178	4
novel circ 0007449	32.6356	0	21.60833	62.74978	0.792904	4
novel circ 0023817	42.84335	4.369006	18.93806	50.18066	0.584764	4
novel circ 0007601	40.63613	0	34.12093	41.38988	0.392512	4
novel circ 0026717	40.08619	0	35.33111	40.58507	0.376394	4
novel circ 0034694	44.01641	20.36453	0	50.91265	0.371557	4
novel circ 0042581	24.79806	0	41.7411	48.53066	0.40048	4
novel circ 0028624	45.81469	0	9.980704	58.79055	0.590034	4
novel circ 0027243	8.215625	0	28.16868	77.66413	0.51731	4
novel circ 0021066	32.58645	0	11.8053	68.36097	0.747476	4
novel circ 0028464	41.01303	0	21.01689	49.67011	0.601951	4
novel circ 0025862	32.6584	25.5088	0	53.37406	0.385039	4
novel circ 0001627	22.23286	0	34.54846	54.06589	0.517427	4
novel circ 0001448	21.44866	0	31.00817	57.69335	0.572024	4
novel circ 0005755	29.73497	0	29.24503	50.84164	0.61854	4
novel circ 0039873	40.77154	0	12.07011	56.52122	0.660412	4
novel circ 0021845	23.36506	0	15.48613	70.08062	0.68912	4
novel circ 0029775	30.98069	0	11.06152	66.51953	0.74175	4
novel circ 0014313	18.54255	13.17705	0	76.47764	0.506855	4
novel circ 0027372	45.21635	0	6.770389	56.07334	0.548332	4
novel circ 0036020	44.84169	0	6.770389	55.64544	0.549092	4
novel circ 0007501	27.87774	22.6855	0	55.79505	0.418857	4
novel circ 0040880	41.81985	0	23.55407	40.08389	0.421273	4
novel circ 0030346	32.43608	0	34.68729	38.25217	0.381998	4
novel circ 0001918	37.46578	20.45872	0	46.83931	0.375172	4
novel circ 0005689	39.41724	0	22.75237	41.89504	0.490653	4
novel circ 0000877	51.44475	0	2.212304	50.33476	0.398151	4
novel circ 0005053	42.62417	0	7.743063	53.2988	0.563396	4
novel circ 0002946	15.6987	10.61699	0	77.04241	0.514417	4
novel circ 0034098	23.85853	0	18.80458	60.1208	0.724766	4
novel circ 0016628	8.621092	0	11.06152	83.0723	0.55468	4
novel circ 0015910	16.54352	32.81439	0	51.90484	0.324377	4
novel circ 0037752	24.27974	27.7404	0	48.42355	0.351221	4
novel circ 0016473	9.896814	0	9.478545	80.74217	0.563719	4
novel circ 0004989	13.24361	0	19.30043	66.65372	0.587493	4
novel circ 0038100	27.55316	10.19435	0	60.52046	0.538877	4
novel circ 0008798	43.57579	0	12.1867	42.45894	0.42863	4
novel circ 0006708	25.97828	0	12.16767	59.52589	0.749711	4
novel circ 0008464	12.45376	22.16143	0	62.86708	0.416547	4
novel circ 0014818	38.59295	10.19435	0	48.68784	0.447053	4
novel circ 0030230	21.92501	0	21.76066	53.33757	0.687588	4
novel circ 0023925	21.34947	0	31.03959	44.50693	0.496152	4
novel circ 0041731	25.15372	0	29.33236	42.37706	0.520203	4
novel circ 0006601	34.36816	0	8.849215	53.5194	0.693086	4
novel circ 0017311	20.59415	0	18.28107	57.68973	0.691974	4
novel circ 0008830	30.89924	22.76035	0	42.44503	0.354703	4
novel circ 0024743	24.61319	28.343	0	42.88764	0.32048	4
novel circ 0041852	28.98307	16.44234	0	50.29821	0.451455	4
novel circ 0018739	29.71057	22.69032	0	42.92405	0.360551	4
novel circ 0007760	17.23571	0	35.39686	42.58404	0.4017	4
novel circ 0006254	29.30861	0	30.07499	35.75881	0.415936	4
novel circ 0033183	29.71987	0	30.69833	34.56102	0.389177	4
novel circ 0029535	30.18582	0	24.25957	40.30398	0.573845	4
novel circ 0031255	31.15971	21.75083	0	41.73254	0.357453	4
novel circ 0043373	19.2586	0	32.00263	43.27649	0.460433	4
novel circ 0006227	24.99621	0	23.71427	45.44666	0.662286	4
novel circ 0040093	47.40162	0	0	46.45293	0.39306	4
novel circ 0040000	17.92859	0	25.47528	50.245	0.585025	4
novel circ 0044321	24.82829	12.72784	0	55.47164	0.505316	4
novel circ 0003062	0	0	25.18434	67.1697	0.471057	4
novel circ 0031332	42.72863	0	8.124467	41.4259	0.413788	4
novel circ 0034876	19.82176	0	28.51242	43.62144	0.520806	4
novel circ 0010564	37.0957	13.01282	0	41.41502	0.384173	4
novel circ 0015587	16.7622	0	32.06132	42.07931	0.437319	4
novel circ 0031927	28.25545	17.47603	0	45.00149	0.41935	4
novel circ 0043271	0	11.97913	16.58307	61.70989	0.43825	4
novel circ 0002102	24.22252	0	8.124467	57.66782	0.715751	4
novel circ 0041356	33.47793	0	18.54743	37.86044	0.541859	4
novel circ 0021496	28.63501	21.76896	0	39.4429	0.349902	4
novel circ 0026553	37.21708	0	9.955367	42.60782	0.534996	4
novel circ 0028847	35.02931	0	5.416311	49.13613	0.609408	4
novel circ 0035651	15.12729	0	13.27382	60.49194	0.632386	4
novel circ 0037616	27.25554	0	9.478545	51.8831	0.75552	4
novel circ 0038326	37.09814	0	15.48613	35.81366	0.432632	4
novel circ 0008528	18.42025	0	30.74336	39.1967	0.437872	4
novel circ 0032187	26.53473	0	0	61.58417	0.636613	4
novel circ 0012214	28.07209	0	4.062233	55.38228	0.679752	4
novel circ 0005923	27.13123	0	19.54836	40.81014	0.696102	4
novel circ 0009530	28.51116	0	26.98636	31.27213	0.392953	4
novel circ 0028604	10.53588	0	15.86753	60.20497	0.578991	4
novel circ 0034959	31.71188	0	18.90017	35.86375	0.533217	4
novel circ 0026644	32.04663	0	10.69914	43.68645	0.664447	4
novel circ 0020588	26.01542	13.01282	0	46.85902	0.476642	4
novel circ 0033523	31.49957	0	17.83191	36.48387	0.558282	4
novel circ 0031018	36.15433	0	5.416311	43.56694	0.532991	4
novel circ 0013156	16.75614	0	18.84326	49.3646	0.65047	4
novel circ 0027109	16.69189	0	0	67.98058	0.598856	4
novel circ 0029281	36.99243	0	12.54907	34.94285	0.413428	4
novel circ 0044044	28.04385	0	0	56.15971	0.631643	4
novel circ 0024443	19.78716	0	28.54107	35.36125	0.43661	4
novel circ 0014811	37.60464	0	0	46.07614	0.496349	4
novel circ 0001378	29.30845	0	13.65523	40.44582	0.700798	4
novel circ 0019329	31.4421	10.78122	0	41.03572	0.438447	4
novel circ 0020354	16.12443	0	18.07983	48.04606	0.650387	4
novel circ 0018608	24.9117	0	5.416311	51.66216	0.703801	4
novel circ 0033031	20.43335	0	5.530759	55.85815	0.677272	4
novel circ 0044263	39.25952	0	4.424607	38.03498	0.402623	4
novel circ 0015644	21.55175	14.37496	0	45.38082	0.460891	4
novel circ 0009479	15.96516	8.738013	0	56.24964	0.524876	4
novel circ 0034538	6.706533	0	5.416311	68.50494	0.55172	4
novel circ 0036004	21.93018	0	24.22089	34.40244	0.513862	4
novel circ 0036733	22.66174	0	16.97368	40.82337	0.759561	4
novel circ 0016288	10.52918	0	16.59228	53.27641	0.581148	4
novel circ 0011105	17.02397	0	13.65523	49.08908	0.694146	4
novel circ 0041904	19.9829	12.75079	0	47.01722	0.482421	4
novel circ 0030321	26.14751	0	17.02159	36.34467	0.674312	4
novel circ 0007910	16.05853	0	4.424607	58.20054	0.632171	4
novel circ 0016595	24.56151	0	11.06152	41.16182	0.785184	4
novel circ 0040461	24.90752	0	0	51.81241	0.634209	4
novel circ 0001198	11.33334	11.65068	0	53.21239	0.477658	4
novel circ 0042275	25.80187	0	8.124467	42.25704	0.731914	4

novel circ 0005436	12.60608	0	15.09549	48.38418	0.621733	4
novel circ 0033952	15.83225	0	12.1867	47.94602	0.685205	4
novel circ 0018371	31.5808	0	15.70639	28.20338	0.380918	4
novel circ 0013540	27.98964	0	0	46.83841	0.604317	4
novel circ 0029854	22.80957	0	18.15817	33.54028	0.641834	4
novel circ 0036505	26.46316	0	0	47.43718	0.617951	4
novel circ 0024240	13.25162	0	8.734767	51.59744	0.637417	4
novel circ 0036392	33.04573	0	0	40.2085	0.493281	4
novel circ 0025226	15.35269	0	21.4782	35.10324	0.546123	4
novel circ 0032553	16.98024	0	0	54.69627	0.618437	4
novel circ 0009167	18.1596	11.08072	0	41.50444	0.485198	4
novel circ 0012556	31.95573	0	0	37.28223	0.47447	4
novel circ 0007440	29.27402	0	2.212304	37.38858	0.538232	4
novel circ 0041608	20.49212	0	4.062233	44.18669	0.694848	4
novel circ 0029907	15.76051	0	14.0176	38.90439	0.711517	4
novel circ 0020318	29.25546	0	4.062233	35.1232	0.527632	4
novel circ 0003293	19.43125	0	9.955367	38.71469	0.791534	4
novel circ 0021971	24.39157	0	11.12846	32.20311	0.66929	4
novel circ 0014794	3.511883	0	13.15937	49.28166	0.524098	4
novel circ 0007390	30.9182	0	0	34.71064	0.456833	4
novel circ 0032728	18.30035	0	0	44.42289	0.635858	4
novel circ 0012032	9.672069	0	0	48.21239	0.583263	4
