# Supplementary figures and images for: Analysis of circular RNA (circRNA) characteristics and identification of key circRNAs in the hypothalamus during sexual maturation in female goats
Source: Anim Biosci. 2025 Jun 24;38(12):2545–57. doi: 10.5713/ab.25.0275 (PMC12580788; doi:10.5713/ab.25.0275)

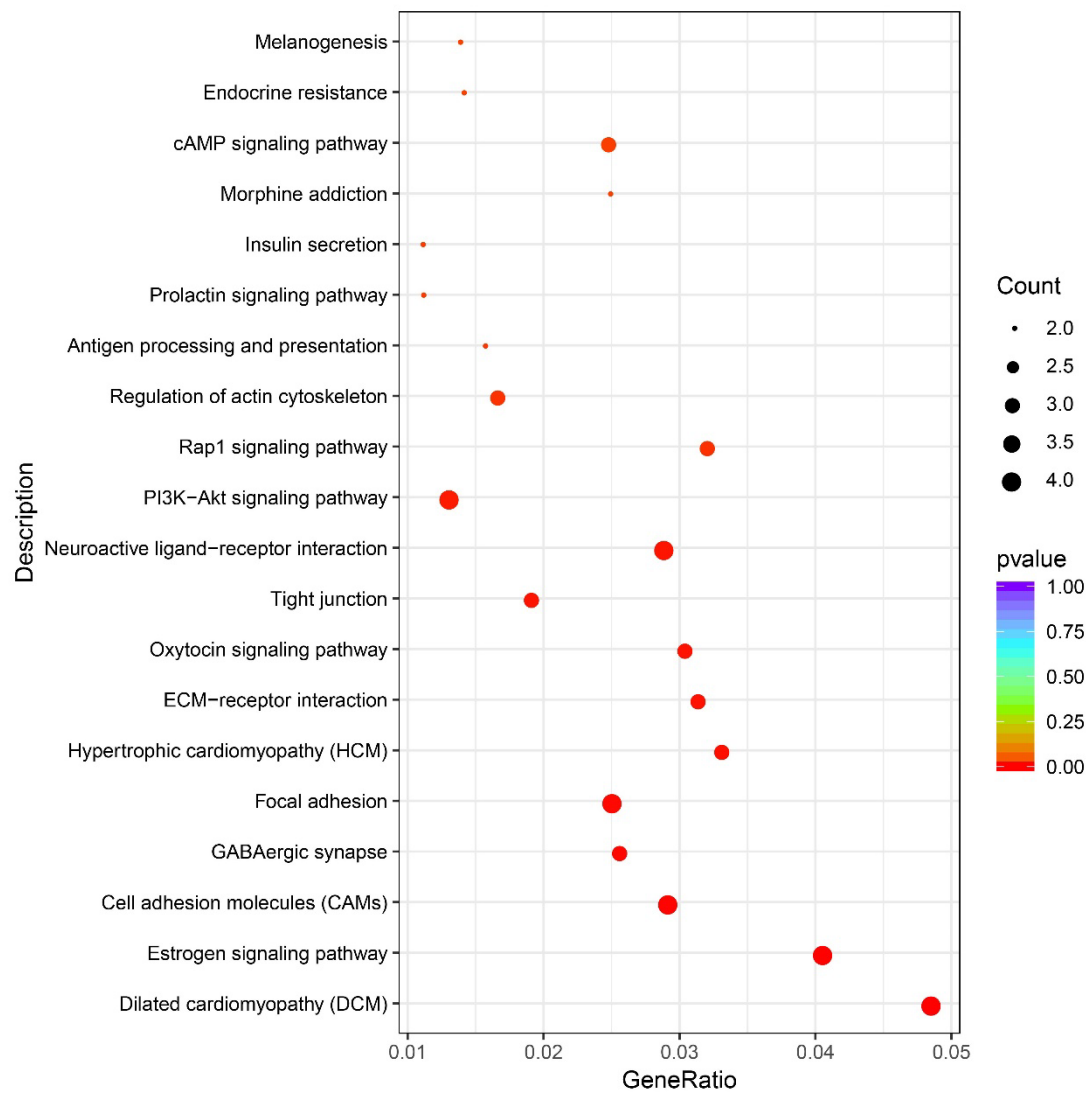

**Supplement 8. KEGG enrichment analysis of mRNAs in the ceRNA network.**

Supplement: Supplementary file 8 [file ab-25-0275-Supplementary-8.pdf]
