# Supplementary material for: Analysis of circular RNA (circRNA) characteristics and identification of key circRNAs in the hypothalamus during sexual maturation in female goats
Source: Anim Biosci. 2025 Jun 24;38(12):2545–57. doi: 10.5713/ab.25.0275 (PMC12580788; doi:10.5713/ab.25.0275)
Supplement: Supplementary file 9 [file ab-25-0275-Supplementary-9.pdf]

Supplement 9. KEGG analysis of mrna in ceRNA network

KEGGID	Description	GeneRatio	BgRatio	pvalue	padj	geneID	geneName	keggID	Count
chx05414	Dilated cardiomyopathy (DCM)	4/82	111/8892	0.0001367	0.0176749	102182568/102	ITGA2B|C	chx:10218	4
chx04915	Estrogen signaling pathway	4/98	106/8892	0.0002638	0.0176749	102174434/102	ITGA2B|C	chx:10217	4
chx04514	Cell adhesion molecules (CAMs)	4/136	147/8892	0.0008728	0.038985	102184383/102	GABBR2|I	chx:10218	4
chx04727	GABAergic synapse	4/155	149/8892	0.001398	0.0401298	102189626/102	OXT|CAC	chx:10218	3
chx04510	Focal adhesion	4/158	13/8892	0.0014974	0.0401298	102168345/102	LRRC4|CL	chx:10216	4
chx05410	Hypertrophic cardiomyopathy (HCM	3/90	133/8892	0.0028536	0.0552763	102176770/102	GABBR2|I	chx:10217	3
chx04512	ECM-receptor interaction	3/95	89/8892	0.0033088	0.0552763	102188907/102	ITGA2B|C	chx:10218	3
chx04921	Oxytocin signaling pathway	3/98	91/8892	0.003602	0.0552763	102174434/102	ITGA2B|T	chx:10217	3
chx04530	Tight junction	4/207	21/8892	0.0038903	0.0552763	102169762/102	ITGA2B|P	chx:10216	3
chx04080	Neuroactive ligand-receptor interacti	3/103	144/8892	0.0041251	0.0552763	102169446/102	PRKCE|AI	chx:10216	4
chx04151	PI3K-Akt signaling pathway	5/375	97/8892	0.0059521	0.0725068	102188173/102	PHLPP2|IT	chx:10218	4
chx04015	Rap1 signaling pathway	2/62	98/8892	0.0161975	0.1808718	102174434/102	ADCY1|HS	chx:10217	3
chx04810	Regulation of actin cytoskeleton	3/178	23/8892	0.0176945	0.1823899	102175283/102	CLDN9|CI	chx:10217	3
chx04612	Antigen processing and presentation	3/190	65/8892	0.020932	0.1918306	102170858/102	IGFBP3|N	chx:10217	2
chx04917	Prolactin signaling pathway	4/356	66/8892	0.0238648	0.1918306	102182568/102	OXT|UCN	chx:10218	2
chx04911	Insulin secretion	4/357	166/8892	0.0240784	0.1918306	102188455/102	ITGA2B|T	chx:10218	2
chx05032	Morphine addiction	2/80	69/8892	0.0257683	0.1918306	102176334/102	HSPA6|LG	chx:10217	2
chx01522	Endocrine resistance	3/211	73/8892	0.0273298	0.1919818	102176770/102	ITGA2B|A	chx:10217	2
chx04916	Melanogenesis	3/215	73/8895	0.028654	0.1919818	102176770/102	ITGA2B|P	chx:10217	2
chx00430	Taurine and hypotaurine metabolism	1/38	20/8948	0.0816776	0.4350513	102175220	GGT7	chx:10217	1
chx00730	Thiamine metabolism	1/38	20/8948	0.0816776	0.4350513	102169601	AK1	chx:10216	1
chx04024	cAMP signaling pathway	3/38	241/8948	0.0817328	0.4350513	108637398/102	OXT/GAB	chx:10863	3
chx04750	Inflammatory mediator regulation of	2/38	113/8948	0.0828373	0.4350513	102180724/102	ADCY1/PI	chx:10218	2
chx04670	Leukocyte transendothelial migratior	2/38	114/8948	0.0840971	0.4350513	102188006/102	CLDN9/CI	chx:10218	2
chx04935	Growth hormone synthesis, secretion	2/38	119/8948	0.0904842	0.4350513	102180724/102	ADCY1/IG	chx:10218	2
chx04380	Osteoclast differentiation	2/38	122/8948	0.0943844	0.4350513	102190607/102	FHL2/FOS	chx:10219	2
chx04071	Sphingolipid signaling pathway	2/38	123/8948	0.0956955	0.4350513	102169016/102	PRKCE/F	chx:10216	2
chx04114	Oocyte meiosis	2/38	123/8948	0.0956955	0.4350513	102180724/102	ADCY1/E	chx:10218	2
chx04611	Platelet activation	2/38	125/8948	0.0983335	0.4350513	102183451/102	ITGA2B/A	chx:10218	2
chx05310	Asthma	1/38	26/8948	0.1048877	0.4504	102168313	FCER1A	chx:10216	1
chx00230	Purine metabolism	2/38	134/8948	0.1104558	0.4607584	102180724/102	ADCY1/A	chx:10218	2
chx04371	Apelin signaling pathway	2/38	139/8948	0.1173561	0.4617097	102180724/102	ADCY1/PI	chx:10218	2
chx04270	Vascular smooth muscle contraction	2/38	145/8948	0.1257793	0.4617097	102180724/102	ADCY1/PI	chx:10218	2
chx04010	MAPK signaling pathway	3/38	294/8948	0.1279757	0.4617097	102190168/102	NGFR/HSI	chx:10219	3
chx04072	Phospholipase D signaling pathway	2/38	148/8948	0.1300457	0.4617097	102180724/102	ADCY1/F	chx:10218	2
chx00500	Starch and sucrose metabolism	1/38	33/8948	0.1312447	0.4617097	102183708	TREH	chx:10218	1
chx05224	Breast cancer	2/38	150/8948	0.1329092	0.4617097	102189484/102	ESR1/TCF	chx:10218	2
chx04215	Apoptosis - multiple species	1/38	35/8948	0.1386354	0.4617097	102190168	NGFR	chx:10219	1
chx04261	Adrenergic signaling in cardiomyocy	2/38	155/8948	0.1401319	0.4617097	102180724/102	ADCY1/C	chx:10218	2
chx04934	Cushing syndrome	2/38	155/8948	0.1401319	0.4617097	102187776/102	TCF7L1/A	chx:10218	2
chx05216	Thyroid cancer	1/38	36/8948	0.1423078	0.4617097	102187776	TCF7L1	chx:10218	1
chx05160	Hepatitis C	2/38	160/8948	0.1474408	0.4679643	102188006/102	CLDN9/CI	chx:10218	2
chx04022	cGMP-PKG signaling pathway	2/38	168/8948	0.1592979	0.4948402	102180724/102	ADCY1/PI	chx:10218	2
chx03410	Base excision repair	1/38	43/8948	0.1675909	0.5005624	102187902	PARP3	chx:10218	1
chx04672	Intestinal immune network for IgA p	1/38	45/8948	0.1746804	0.5005624	102180190	ITGB7	chx:10218	1
chx03050	Proteasome	1/38	46/8948	0.178203	0.5005624	100860840	PSMA6	chx:10086	1
chx04930	Type II diabetes mellitus	1/38	46/8948	0.178203	0.5005624	102169016	PRKCE	chx:10216	1
chx04360	Axon guidance	2/38	181/8948	0.1789346	0.5005624	102171839/10	LRRC4/PA	chx:10217	2
chx00514	Other types of O-glycan biosynthesis	1/38	47/8948	0.181711	0.5005624	102187411	B3GLCT	chx:10218	1
chx05165	Human papillomavirus infection	3/38	354/8948	0.1891162	0.5113141	102187776/102	TCF7L1/IT	chx:10218	3
chx04961	Endocrine and other factor-regulated	1/38	52/8948	0.1990335	0.5189088	102189484	ESR1	chx:10218	1
chx05144	Malaria	1/38	52/8948	0.1990335	0.5189088	102169438	ACKR1	chx:10216	1
chx05205	Proteoglycans in cancer	2/38	205/8948	0.2160853	0.5274436	102189484/102	ESR1/PLA	chx:10218	2
chx05213	Endometrial cancer	1/38	58/8948	0.2193495	0.5274436	102187776	TCF7L1	chx:10218	1
chx05207	Chemical carcinogenesis - receptor a	2/38	210/8948	0.2239297	0.5274436	102189484/102	ESR1/AD	chx:10218	2
chx04923	Regulation of lipolysis in adipocytes	1/38	60/8948	0.2260094	0.5274436	102180724	ADCY1	chx:10218	1
chx05134	Legionellosis	1/38	60/8948	0.2260094	0.5274436	102185412	HSPA6	chx:10218	1
chx00480	Glutathione metabolism	1/38	63/8948	0.2358955	0.5274436	102175220	GGT7	chx:10217	1
chx04929	GnRH secretion	1/38	63/8948	0.2358955	0.5274436	102186689	GABBR2	chx:10218	1
chx05217	Basal cell carcinoma	1/38	63/8948	0.2358955	0.5274436	102187776	TCF7L1	chx:10218	1
chx04664	Fc epsilon RI signaling pathway	1/38	65/8948	0.2424179	0.5274436	102168313	FCER1A	chx:10216	1
chx04913	Ovarian steroidogenesis	1/38	65/8948	0.2424179	0.5274436	102180724	ADCY1	chx:10218	1
chx04927	Cortisol synthesis and secretion	1/38	65/8948	0.2424179	0.5274436	102180724	ADCY1	chx:10218	1
chx04720	Long-term potentiation	1/38	66/8948	0.2456587	0.5274436	102180724	ADCY1	chx:10218	1
chx05221	Acute myeloid leukemia	1/38	68/8948	0.2520999	0.5334287	102187776	TCF7L1	chx:10218	1
chx05211	Renal cell carcinoma	1/38	70/8948	0.2584875	0.5334622	100860973	PAK6	chx:10086	1
chx05166	Human T-cell leukemia virus 1 infec	2/38	235/8948	0.2634464	0.5334622	102180724/102	ADCY1/E	chx:10218	2
chx04014	Ras signaling pathway	2/38	238/8948	0.2682063	0.5334622	102190168/10	NGFR/PA	chx:10219	2
chx04918	Thyroid hormone synthesis	1/38	76/8948	0.2773332	0.5334622	102180724	ADCY1	chx:10218	1
chx04721	Synaptic vesicle cycle	1/38	78/8948	0.2835109	0.5334622	102182144	SNAP25	chx:10218	1
chx04971	Gastric acid secretion	1/38	79/8948	0.2865804	0.5334622	102180724	ADCY1	chx:10218	1
chx04742	Taste transduction	1/38	84/8948	0.3017369	0.5334622	102186689	GABBR2	chx:10218	1
chx04012	ErbB signaling pathway	1/38	85/8948	0.3047304	0.5334622	100860973	PAK6	chx:10086	1
chx04115	p53 signaling pathway	1/38	85/8948	0.3047304	0.5334622	102174541	IGFBP3	chx:10217	1
chx01232	Nucleotide metabolism	1/38	86/8948	0.3077113	0.5334622	102169601	AK1	chx:10216	1
chx04540	Gap junction	1/38	88/8948	0.313636	0.5334622	102180724	ADCY1	chx:10218	1
chx04211	Longevity regulating pathway	1/38	89/8948	0.3165797	0.5334622	102180724	ADCY1	chx:10218	1
chx05210	Colorectal cancer	1/38	89/8948	0.3165797	0.5334622	102187776	TCF7L1	chx:10218	1
chx04912	GnRH signaling pathway	1/38	91/8948	0.3224305	0.5334622	102180724	ADCY1	chx:10218	1
chx04260	Cardiac muscle contraction	1/38	92/8948	0.3253375	0.5334622	102172403	CACNG3	chx:10217	1
chx04520	Adherens junction	1/38	92/8948	0.3253375	0.5334622	102187776	TCF7L1	chx:10218	1
chx04914	Progesterone-mediated oocyte matur	1/38	92/8948	0.3253375	0.5334622	102180724	ADCY1	chx:10218	1
chx05020	Prion disease	2/38	275/8948	0.326767	0.5334622	102185412/10	HSPA6/PS	chx:10218	2
chx04610	Complement and coagulation cascad	1/38	94/8948	0.3311152	0.5334622	102186624	PLAUR	chx:10218	1
chx04970	Salivary secretion	1/38	94/8948	0.3311152	0.5334622	102180724	ADCY1	chx:10218	1
chx05215	Prostate cancer	1/38	97/8948	0.3396915	0.5334622	102187776	TCF7L1	chx:10218	1
chx04666	Fc gamma R-mediated phagocytosis	1/38	98/8948	0.3425264	0.5334622	102169016	PRKCE	chx:10216	1
chx04713	Circadian entrainment	1/38	98/8948	0.3425264	0.5334622	102180724	ADCY1	chx:10218	1
chx05222	Small cell lung cancer	1/38	98/8948	0.3425264	0.5334622	102183451	ITGA2B	chx:10218	1
chx04350	TGF-beta signaling pathway	1/38	101/8948	0.3509602	0.5334622	100860914	FST	chx:10086	1
chx04660	T cell receptor signaling pathway	1/38	101/8948	0.3509602	0.5334622	100860973	PAK6	chx:10086	1
chx04640	Hematopoietic cell lineage	1/38	104/8948	0.3592886	0.5334622	102183451	ITGA2B	chx:10218	1
chx04928	Parathyroid hormone synthesis, secre	1/38	104/8948	0.3592886	0.5334622	102180724	ADCY1	chx:10218	1
chx04933	AGE-RAGE signaling pathway in di	1/38	104/8948	0.3592886	0.5334622	102169016	PRKCE	chx:10216	1
chx04972	Pancreatic secretion	1/38	106/8948	0.364783	0.5334622	102180724	ADCY1	chx:10218	1
chx04976	Bile secretion	1/38	109/8948	0.3729386	0.5334622	102180724	ADCY1	chx:10218	1
chx04724	Glutamatergic synapse	1/38	111/8948	0.3783189	0.5334622	102180724	ADCY1	chx:10218	1
chx04725	Cholinergic synapse	1/38	111/8948	0.3783189	0.5334622	102180724	ADCY1	chx:10218	1
chx04931	Insulin resistance	1/38	113/8948	0.3836543	0.5334622	102169016	PRKCE	chx:10216	1
chx05142	Chagas disease	1/38	113/8948	0.3836543	0.5334622	102180724	ADCY1	chx:10218	1
chx05145	Toxoplasmosis	1/38	113/8948	0.3836543	0.5334622	102185412	HSPA6	chx:10218	1
chx05146	Amoebiasis	1/38	115/8948	0.3889451	0.5357169	102180724	ADCY1	chx:10218	1
chx04722	Neurotrophin signaling pathway	1/38	122/8948	0.4071169	0.5503618	102190168	NGFR	chx:10219	1
chx04919	Thyroid hormone signaling pathway	1/38	122/8948	0.4071169	0.5503618	102189484	ESR1	chx:10218	1
chx04060	Cytokine-cytokine receptor interactic	2/38	339/8948	0.4249647	0.567065	102191371/102	TNFSF12/	chx:10219	2
chx04926	Relaxin signaling pathway	1/38	130/8948	0.4272408	0.567065	102180724	ADCY1	chx:10218	1
chx03040	Spliceosome	1/38	138/8948	0.446699	0.582304	102185412	HSPA6	chx:10218	1
chx04142	Lysosome	1/38	138/8948	0.446699	0.582304	102191183	LGMN	chx:10219	1
chx05162	Measles	1/38	143/8948	0.4585318	0.5851028	102185412	HSPA6	chx:10218	1
chx05418	Fluid shear stress and atherosclerosi	1/38	143/8948	0.4585318	0.5851028	102183451	ITGA2B	chx:10218	1
chx05017	Spinocerebellar ataxia	1/38	144/8948	0.4608687	0.5851028	100860840	PSMA6	chx:10086	1
chx04723	Retrograde endocannabinoid signalir	1/38	146/8948	0.4655129	0.5851701	102180724	ADCY1	chx:10218	1
chx04120	Ubiquitin mediated proteolysis	1/38	149/8948	0.4724063	0.5851701	102173985	UBE2Q1	chx:10217	1
chx04936	Alcoholic liver disease	1/38	150/8948	0.4746848	0.5851701	102187776	TCF7L1	chx:10218	1
chx04210	Apoptosis	1/38	151/8948	0.4769537	0.5851701	102187902	PARP3	chx:10218	1
chx05226	Gastric cancer	1/38	153/8948	0.481463	0.58578	102187776	TCF7L1	chx:10218	1
chx04390	Hippo signaling pathway	1/38	157/8948	0.4903682	0.5907948	102187776	TCF7L1	chx:10218	1
chx05206	MicroRNAs in cancer	1/38	160/8948	0.4969493	0.5907948	102169016	PRKCE	chx:10216	1
chx01240	Biosynthesis of cofactors	1/38	163/8948	0.5034475	0.5907948	102169601	AK1	chx:10216	1
chx04110	Cell cycle	1/38	163/8948	0.5034475	0.5907948	102177959	ESPL1	chx:10217	1
chx04145	Phagosome	1/38	166/8948	0.509864	0.5907948	102168670	PLA2R1	chx:10216	1
chx04218	Cellular senescence	1/38	166/8948	0.509864	0.5907948	102174541	IGFBP3	chx:10217	1
chx04141	Protein processing in endoplasmic re	1/38	171/8948	0.5203792	0.595926	102185412	HSPA6	chx:10218	1
chx04310	Wnt signaling pathway	1/38	172/8948	0.5224557	0.595926	102187776	TCF7L1	chx:10218	1
chx05225	Hepatocellular carcinoma	1/38	174/8948	0.5265825	0.5959771	102187776	TCF7L1	chx:10218	1
chx05152	Tuberculosis	1/38	186/8948	0.5506238	0.6183929	102168670	PLA2R1	chx:10216	1
chx04062	Chemokine signaling pathway	1/38	189/8948	0.5564459	0.6201611	102180724	ADCY1	chx:10218	1
chx04613	Neutrophil extracellular trap formati	1/38	206/8948	0.588076	0.6465675	102183451	ITGA2B	chx:10218	1
chx04630	JAK-STAT signaling pathway	1/38	209/8948	0.593425	0.6465675	102169142	CISH	chx:10216	1
chx05167	Kaposi sarcoma-associated herpesvir	1/38	209/8948	0.593425	0.6465675	102187776	TCF7L1	chx:10218	1
chx05417	Lipid and atherosclerosis	1/38	230/8948	0.6290195	0.6770069	102185412	HSPA6	chx:10218	1
chx05170	Human immunodeficiency virus 1 in	1/38	231/8948	0.6306365	0.6770069	100860973	PAK6	chx:10086	1
chx04020	Calcium signaling pathway	1/38	238/8948	0.6417651	0.6789882	102180724	ADCY1	chx:10218	1
chx04714	Thermogenesis	1/38	240/8948	0.6448843	0.6789882	102180724	ADCY1	chx:10218	1
chx05163	Human cytomegalovirus infection	1/38	241/8948	0.646434	0.6789882	102180724	ADCY1	chx:10218	1
chx04144	Endocytosis	1/38	255/8948	0.6674499	0.6960549	102185412	HSPA6	chx:10218	1

chx05132	Salmonella infection	1/38	262/8948	0.6774967	0.7015214	102187776	TCF7L1	chx:10218[illegible]	1
chx05012	Parkinson disease	1/38	274/8948	0.6940363	0.7135866	100860840	PSMA6	chx:10086[illegible]	1
chx05016	Huntington disease	1/38	310/8948	0.7388521	0.7543525	100860840	PSMA6	chx:10086[illegible]	1
chx05014	Amyotrophic lateral sclerosis	1/38	376/8948	0.8050002	0.8161808	100860840	PSMA6	chx:10086[illegible]	1
chx05010	Alzheimer disease	1/38	390/8948	0.8167684	0.8224013	100860840	PSMA6	chx:10086[illegible]	1
chx05022	Pathways of neurodegeneration - mu[illegible]	1/38	480/8948	0.8774973	0.8774973	100860840	PSMA6	chx:10086[illegible]	1
