# Supplementary material for: Analysis of circular RNA (circRNA) characteristics and identification of key circRNAs in the hypothalamus during sexual maturation in female goats
Source: Anim Biosci. 2025 Jun 24;38(12):2545–57. doi: 10.5713/ab.25.0275 (PMC12580788; doi:10.5713/ab.25.0275)
Supplement: Supplementary file 10 [file ab-25-0275-Supplementary-10.pdf]

Supplement 10. circRNA junction sequence information

circRNA name	Location	Sequence
novel_circ_0005937	chr NC_030810.1:27240437-27241192	AATTCCTCACAGGCGAAGGACATTTGAagGTCTCCTGAATGATGCAACTGTAGGTATT
novel_circ_0002274	chr NC_030808.1:76396785-76401607	atatcAAACCAAACATCACTTGGTATATGAACGCTGTGATGACTGGGGACTAGATACT
novel_circ_0025302	chr NC_030819.1:75395955-75401305	ATGTAAAGGTGGAATTTTCAGAAAAAGAGGATATGTGAATACCATGAAAAAAACTATG
novel_circ_0030183	chr NC_030823.1:36162081-36213902	CACAAGAGATGTCATAATCAAGGAAACACACCCTGAAAACCAAACCTTGAGAAAAGAT
novel_circ_0006250	chr NC_030810.1:39599842-39646715	CTAAAACATACTAAAGAATGGAGTTTAAGTAGACCCACATTCTGGCAAGCCCTGTATA
